# Impact of climate factors on height growth of *Pinus sylvestris* var. *mongolica*

**DOI:** 10.1371/journal.pone.0213509

**Published:** 2019-03-11

**Authors:** Yanping Zhou, Zeyong Lei, Fengyan Zhou, Yangang Han, Deliang Yu, Yansong Zhang

**Affiliations:** 1 Department of Soil and Water Conservation and Desertification Combating, College of Environmental Science and Engineering, Liaoning Technical University, Fuxin, Liaoning, People’s Republic of China; 2 Liaoning Institute of Sandy Land Control and Utilization, Fuxin, Liaoning, People’s Republic of China; 3 Key Laboratory of Forest Ecology and Management, Institute of Applied Ecology, Chinese Academy of Sciences, Shenyang, People’s Republic of China; 4 University of Chinese Academy of Sciences, Beijing, People’s Republic of China; Chinese Academy of Sciences, CHINA

## Abstract

Tree height growth is sensitive to climate change; therefore, incorporating climate factors into tree height prediction models can improve our understanding of this relationship and provide a scientific basis for plantation management under climate change conditions. Mongolian pine (*Pinus sylvestris* var. *mongolica*) is one of the most important afforestation species in Three-North Regions in China. Yet our knowledge on the relationship between height growth and climate for Mongolian pine is limited. Based on survey data for the dominant height of Mongolian pine and climate data from meteorological station, a mixed-effects Chapman-Richards model (including climate factors and random parameters) was used to study the effects of climate factors on the height growth of Mongolian pine in Zhanggutai sandy land, Northeast China. The results showed that precipitation had a delayed effect on the tree height growth. Generally, tree heights increased with increasing mean temperature in May and precipitation from October to April and decreased with increasing precipitation in the previous growing season. The model could effectively predict the dominant height growth of Mongolian pine under varying climate, which could help in further understanding the relationship between climate and height growth of Mongolian pine in semiarid areas of China.

## Introduction

Forests are important components of terrestrial ecosystems. They cover ~30% of the global land surface and play an important role in maintaining global carbon balance and biodiversity [1]. To improve the ecological, economic, and social benefits of forests and realize the sustainable development, it is necessary to understand the growth law of trees and to accurately predict tree growth. Both height and diameter at breast height are affected by the genetic characteristics of trees and local environmental factors [2]. Climate is the most influential factor affecting the growth and distribution of trees [3]. As the structural function and growth patterns of forests have been altered by global climate change, an enhanced tree growth prediction model can help provide a basis for sustainable forest management. New growth models that incorporate climate attributes as a pivotal part of independent variables are necessary [4]. The growth–climate relationship is normally assessed through an analysis of secondary growth via diverse tree-ring proxies, but the relationship between primary (height) growth and climate has rarely been studied because of difficulty in data collection [3,5]. Given that tree height is an important indicator of forest dynamics, detecting and quantifying the relationship between tree growth and the surrounding environmental or climatic factors can help determine the impact of external environments on tree growth. This approach also provides a basis for more effective predictions of forest dynamics under future changing climate scenarios to better inform plantation management. Dominant tree height, for example, has been used to calculate the site index for even-aged and pure forests [6]. The dominant height growth model with climate factors is used to help predict the site index under future climate change and to analyze the adaptation strategies of trees [7].

Statistical growth models using climate factors can quantitatively describe the complex relationship between tree height and environmental conditions, which may more accurately reflect and predict the impact of climate change on tree and stand growth and survival [8–10]. Messaoud and Chen [11] reported that the response of height growth to recent climate change varies with tree species and the spatial environment, concluding that the height growths of both *Populus tremuloides* and *Picea mariana* are positively related to temperature variables at the regional scale. Although the height growth of *Populus tremuloides* was not significantly related to any temporal variables, *Picea mariana* increased with later stand establishment date, higher average maximum summer temperature between May and August, and higher atmospheric CO_2_ concentrations [11]. Ferraz Filho [12] reported that the addition of climatic variables (precipitation and solar radiation) to the inclination parameters of the Chapman-Richards model resulted in more precise estimates of dominant height. Leites et al. [13] concluded that the three-year height growth of *Pseudototsuga menziesii* was most sensitive to the mean temperature in the coldest month. Scolforo [9] incorporated the mean monthly precipitation and temperature into the Chapman-Richards model to predict the dominant height growth of *Eucalyptus grandis*, where both factors composed the model’s asymptote modifier. In addition, introducing both random parameters and climate factors to growth models can help simulate tree height dynamics, distinguish differences between sites [14], and describe the response of height growth–climate relationship [13].

The current Three-North Forest Shelterbelt Program, which started in 1978 to combat ongoing desertification, is the largest forestry project in China and even worldwide. The widely planted Mongolian pine (*Pinus sylvestris* var. *mongolica*) was first introduced to Zhanggutai, Liaoning Province, China (N42°43′–43°20′, E122°22′–123°22′) from its natural habitat in Honghuaerji (N47°35′–48°36′, E118°58′–120°32′) in the 1950s to establish sand-fixing plantations. It is one of the most important afforestation species distributed sporadically or centrally in wide climatic zones. Adapting to climatic change is crucial for tree survival and growth, and growth will respond differently to varying environmental conditions, especially when trees are introduced to different geographic regions [11,13,15]. Changes in environmental conditions may also causes variations in the genetic characteristics, morphology, and growth characteristics of trees [10], as has been observed for Mongolian pine in different regions [16]. When this species was shifted 5° southward from its hometown in Honghuaerji to Zhanggutai, where the mean annual precipitation and the mean annual, lowest, and highest temperatures varied significantly [17], it showed accelerated growth and an earlier maturity period along with a shorter life span (~60 years) [18]. Thus, clearly understanding the relationship between climate and Mongolian pine growth is significant for guiding its introduction, cultivation, management, and harvest. Nevertheless, a quantitative climate–height growth model for Mongolian pine plantations requires further exploration. To our knowledge, no studies have addressed the climate–height growth model for Mongolian pine plantations in semi-arid areas in northern China.

The main objective of this study is to develop a statistical model that reflects the impact of climate factors on the height growth of Mongolian pine trees for different stands.

## Materials and methods

### Study area

The present study was conducted at the Liaoning Province Sand-Fixation and Afforestation Research Institute, southern Horqin sandy land, Northeast China. The annual average temperature and precipitation in this region over the past 40 years (1974–2015) were 6.97°C and 482.9 mm, respectively, with nearly 67.0% of the precipitation occurring from June to August. Early frost is known to appear toward the end of September or early October, and late frost appears in mid or late April [19]. The annual frost-free period lasts for 150 to 160 days [20]. Representative plants include *Acer mono*, *Crataegus pinnatifida* var. *major*, *Ulmus pumila*, *Ulmus macrocarpa*, *Armeniaca sibirica*, *Lespedeza bicolor*, and *Cleistogenes chinensis* [21].

### Stand data

**Fig 1:**
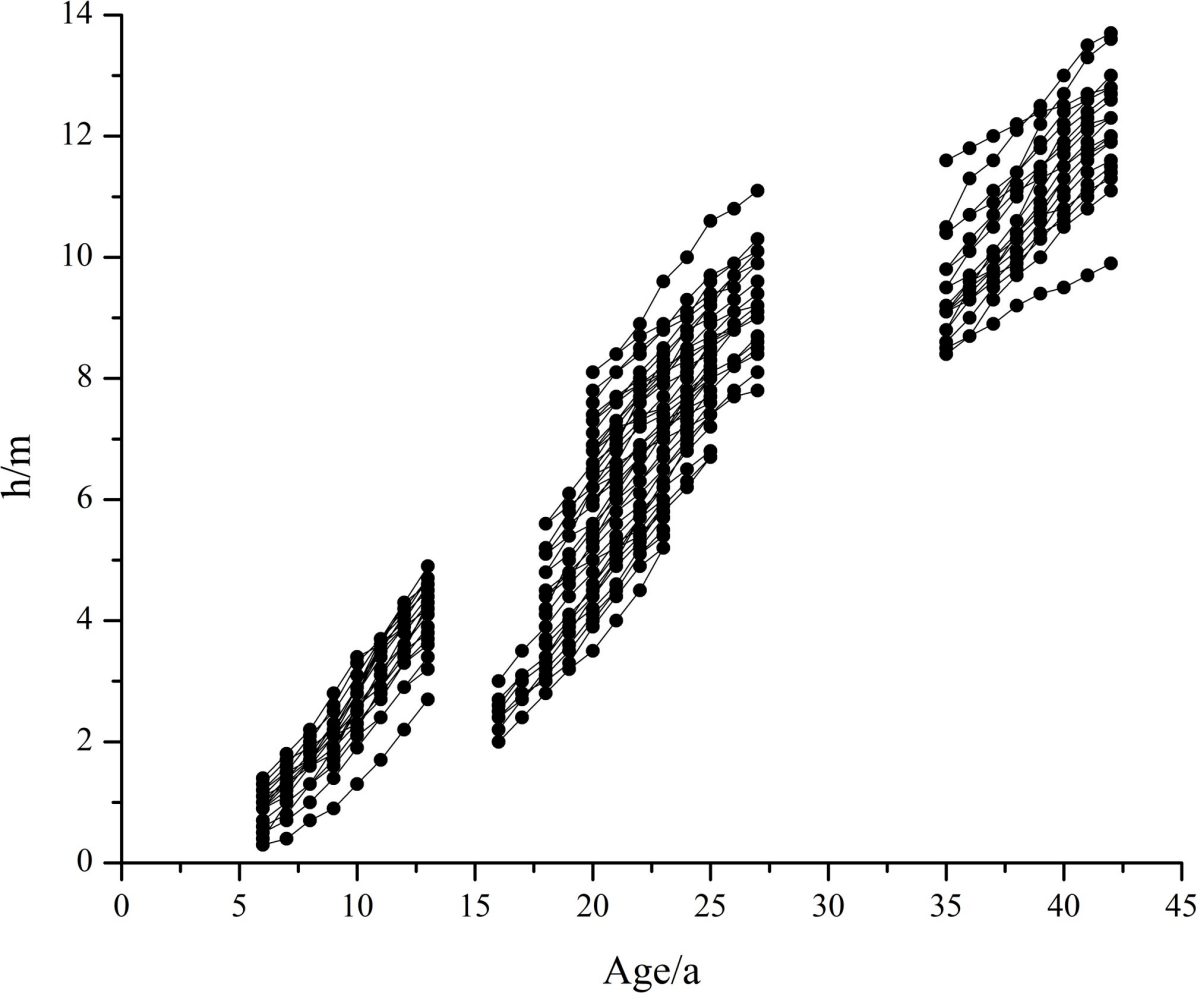
Relationship between height and age of mongolian pine.

The data for the present study were collected in April 2016 from temporary sample plots. The sample sites were selected by the following three criteria: (1) they should, to the extent possible, cover different stand ages of Mongolian pine; (2) they should be pure stands and cover different site conditions; and (3) the distance between each sample sites should > 100 m and sites at the edges of roads and farmlands should be avoided. Thirty sites (20 × 20 m each) with stand ages ranging from 13 to 62 yrs were sampled. The sites included flat sand lands (7 plots) and dunes with different slope directions and positions. The top, middle, and bottom dunes consisted of 7, 10, and 6 plots, respectively. All standing and living trees in each sample plot were measured for total height (H) in the last 8 years, over-bark diameter at breast height (DBH), and crown width (CW). The height was measured using an infrared dendrometer (Criterion™ RD1000, Wuhan, China) with a precision of 0.1 m, and the over-bark DBH and CW were measured using a tape with a precision of 0.1 cm. The stand ages for Mongolian pine plantations were obtained from the records of afforestation units. Because the height of dominant trees is not affected by the stand density [22], the height growth of Mongolian pine was analyzed by considering the dominant trees. The average height of 100 trees with the largest DBH per hectare was considered as the dominant height (TH) [23,24]. Therefore, four trees with the largest DBH were selected as the dominant trees on each sample plot. Climate data could only be obtained from 1974 to 2015, and tree height growth is a cumulative result since it has been planted. As a result, the climate data of the tree growth period were averaged to correspond to the tree height data, and some stands with ages not covered by the climate data interval were excluded for analysis. Finally, the survey data (Fig 1) were divided into model fitting data (the height of 68 dominant trees from 17 sample plots for eight consecutive years [2008–2015], ~ 80%) and model validation data (heights of 16 dominant trees from 4 sample plots for the same years ~ 20%; Table 1, Table 1 is calculated by S1 and S3 Tables). The survey data collected in April 2016 represented the cumulative growth in 2015 because height growth initiated during spring [25].

**Table 1:** Summary statistics of stand variables.

Data type	Statistic	Age (yr)	TH (m)	DBH (cm)	CW(m)
Aggregate data	Min	13	2.7	9.49	2.0
	Max	42	13.7	26.99	6.5
	Mean ± SE	26 ± 2.3	8.1 ± 0.33	17.57 ± 0.503	4.17 ± 0.102
Model fitting data	Min	13	3.7	9.55	2.83
	Max	42	13.7	26.99	6.5
	Mean ± SE	27 ± 2.5	8.3 ± 0.36	17.73 ± 0.582	4.28 ± 0.111
Model validation data	Min	13	2.7	9.49	2.00
	Max	42	12.3	21.23	4.73
	Mean ± SE	26 ± 6.0	7.3 ± 0.84	16.86 ± 0.927	3.70 ± 0.216

### Climate data

Climate data from 1974 to 2015 (Table 2, see S4 Table for origin data) were obtained from the Zhanggutai Meteorological Station, which is <15 km from the sample plots. We used the following climate factors:

- Mean annual temperature (MAT,°C): monthly average temperature averaged over the year;
- Annual precipitation (P, mm): sum of the monthly precipitation for the year;
- Mean temperature in the coldest month (MTCM,°C): average temperature in January;
- Mean temperature in the warmest month (MTWM,°C): average temperature in July;
- Mean temperature in May (MTM,°C): average temperature in May;
- Precipitation in May (PM, mm): sum of the daily precipitation in May;
- Growing season temperature (GST,°C): monthly temperature averaged from May to September;
- Growing season precipitation (GSP, mm): sum of the monthly precipitation from May to September;
- Growing degree-days above 5°C (GDD,°C): sum of the difference between daily average temperature (> 5°C) and 5°C from May to September;
- Aridity index of Kira (AIK, mm°C^-1^): the ratio of annual precipitation (P) to warmth index (WI, sum of the monthly mean temperature above 5°C) [26]. When WI = 0–100°C per month, AIK = P/(WI + 20); when WI > 100°C per month, AIK = P/(WI + 140);
- Precipitation from the previous October to the current April (PNP, mm): sum of precipitation from the previous October to current April;
- Precipitation in the previous growing season (PGP, mm): sum of precipitation from previous May to previous September;
- Annual precipitation in the previous year (PL, mm): total precipitation in the previous year.

**Table 2:** Summary statistics of climate variables.

Project	MAT (°C)	P (mm)	MTCM (°C)	MTWM (°C)	MTM (°C)	PM (mm)	GST (°C)	GSP (mm)	GDD (°C)	AIK (mm°C^-1^)	PNP (mm)	PGP (mm)	PL (mm)
Min	5.3	285.9	−19.11	21.91	14.31	0	18.88	166.6	1982.35	2.78	18.15	166.6	285.9
Max	8.99	713.2	−8.02	26.13	18.83	105.3	21.9	641.9	2487.7	7.12	178.4	641.9	713.2
Mean	6.97	482.9	−12.98	23.55	16.31	39.6	20.05	405.8	2212.64	4.73	77.2	409.9	487.1
SE	0.12	19	0.37	0.16	0.17	3.6	0.11	17.4	21.49	0.2	5.8	17.1	18.7

### Basic model

The Chapman-Richards model was used as the basic dominant height growth model in this regard (more details about the model screening can be found in Zhou et al. [27]). The statistical expectation of the Chapman-Richards model can be expressed as:

$$h=a_{1}{\left[1‑{\exp(‑a}_{2}t)\right]}^{a_{3}}+\varepsilon$$ (1)

where, h and t are the dominant height (m) and age (a), respectively; a_1_ is the asymptote parameter that denotes the asymptotic value of the dominant height; a_2_ and a_3_ are the rate and shape parameters, respectively; and ε is the random error term.

### Generalized model

After establishing the basic model, the correlation between 13 climatic factors was analyzed to select a subset of climatic factors that were not significantly correlated with each other (Table 3, see S4 Table for origin data). To reduce the multicollinearity among variables and ensure high accuracy of the model, MTCM, MTM, PGP, and PNP were selected as the candidate climate factors. The factors or factor combinations were incorporated into the model using a power function form to develop the generalized dominant height model [14,23,28]. The final function was determined based on the goodness-of-fit indices: Akaike’s information criterion (AIC), Bayesian information criterion (BIC), and log-likelihood (LL). Finally, MTM, PNP, and PGP were added to the basic model:

$$h={\mathrm{MTM}}^{a_{4}}{\mathrm{PNP}}^{a_{5}}{\mathrm{PGP}}^{a_{6}}{{[1‑\exp(‑a}_{2}t)]}^{a_{3}}+\varepsilon$$ (2)

where a_2_–a_6_ are the parameters estimated.

**Table 3:** Correlations among climate factors.

	MAT	P	MTCM	MTWM	MTM	PM	GST	GSP	GDD	AIK	PGP	PNP	PL
**MAT**	1												
**P**	−0.08	1											
**MTCM**	0.593**	−0.04	1										
**MTWM**	0.28	−0.07	−0.08	1									
**MTM**	0.335*	−0.26	0.09	0.21	1								
**PM**	0.05	0.23	0.08	0.02	−0.376*	1							
**GST**	0.531**	−0.23	0.05	0.750**	0.516**	−0.06	1						
**GSP**	−0.09	0.944**	−0.10	−0.07	−0.375*	0.21	–0.28	1					
**GDD**	0.689**	−0.17	0.29	0.587**	0.683**	−0.15	0.818**	−0.26	1				
**AIK**	−0.20	0.984**	−0.05	−0.17	−0.313*	0.22	−0.363*	0.929**	−0.29	1			
**PGP**	−0.09	0.07	0.16	−0.312*	−0.24	−0.05	−0.365*	0.08	−0.12	0.11	1		
**PNP**	0.15	0.320*	0.12	0.04	0.24	0.06	0.16	0.13	0.336*	0.28	0.11	1	
**PL**	−0.03	0.18	0.13	−0.24	−0.17	−0.05	−0.27	0.18	−0.01	0.20	0.942**	0.25	1

*Significantly correlated at the 0.05 level (bilateral)

**significantly correlated at the 0.01 level (bilateral).

### Nonlinear mixed-effects model

As the height growth varied from one stand to another, we established a one-level (plot-level random effect) nonlinear mixed-effects model for dominant height. The general expression for the mixed-effects model compared the AIC and BIC values of different combinations of mixed models [29,30]:

$$h_{ij}=MTM^{a_{4}}{\mathrm{PNP}}^{a_{5}}{\mathrm{PGP}}^{a_{6}}{{[1‑e}^{{‑(a}_{2}+u_{i}{)t}_{\mathrm{ij}}}]}^{a_{3}}{+\varepsilon }_{\mathrm{ij}}$$ (3)

where, h_ij_ is the dominant height (m) at age t_ij_ (a) of the plot i; ε_ij_ is the random error, which was assumed to be normally distributed as ε_ij_ ~ N(0, R_i_); a_2_–a_6_ are the fixed effects parameters; and u_i_ is the random effects parameters with a variance-covariance matrix (D), that is, u_i_ ~ N (0, D).

The within-plot variance-covariance matrix (R_i_) was determined as:

$$R_{i}=\sigma^{2}G_{i}^{0.5}\Gamma_{i}G_{i}^{0.5}$$ (4)

where, σ^2^ is the scaling factor for error dispersion such that σ^2^ is equal to the residual variance of the model; G_i_ is the diagonal matrix to describe heteroscedasticity of plot i; and Γ_i_ is the autocorrelation matrix of plot i.

Three alternative variance functions (power function, exponential function, and constant plus power function) were tested for eliminating heteroscedasticity.

$$Var(\varepsilon_{ij})=\sigma^{2}t_{ij}{}^{2\delta }$$ (5)

$$Var(\varepsilon_{ij})=\sigma^{2}exp\left(2\delta t_{ij}\right)$$ (6)

$$Var(\varepsilon_{ij})=\sigma^{2}{\left(\delta_{1}+t_{ij}{}^{\delta 2}\right)}^{2}$$ (7)

where: ε_ij_ is the residual vector; *σ*^2^ is the scaling factor for error dispersion, which is equal to the estimated residual variance; t_ij_ is the tree age; and *δ*, *δ*_1_, and *δ*_2_ are the parameters estimated.

The within-sample-plot autocorrelations of the residuals were considered by using four common correlation structures [AR(1), MA(1), ARMA(1,1) and CS]. When using the autocorrelation structure, the improvement effect for the model was not good;thus, the problem of autocorrelation was not considered.

The among-plot variance-covariance matrix was determined as:

$$D=[\sigma_{i}{}^{2}]$$ (8)

where, D is the among-plot variance-covariance matrix and σ_i_^2^ is the variances of u_i_.

### Estimation of model parameters

The fixed-effects model utilized the least squares method to estimate the parameters with calculations carried out using the *nls* package in the R software. The nonlinear mixed-effects model utilized the maximum likelihood estimation to estimate the parameters using calculations carried out with the *nlme* package in the R software [31].

### Model evaluation and validation

The prediction performance of the model was evaluated through comparison with the validation data. Predictions were made directly with the fixed-effects models, requiring the calculation of random parameters (u_i_) based on the empirical best linear unbiased prediction theory [32]:

$$u_{i}\approx \hat{D}{\hat{Z}}_{i}^{T}{\left({\hat{Z}}_{i}\hat{D}{\hat{Z}}_{i}^{T}+{\hat{R}}_{i}\right)}^{‑1}{\hat{e}}_{i}$$ (9)

where, $\hat{D}$ is the estimated variance-covariance matrix for the random-effects u_i_; ${\hat{R}}_{i}$ is the estimated variance-covariance matrix for the error term; ${\hat{e}}_{i}$ is the residual vector with predicted value estimated by the model including only fixed effects; and ${\hat{Z}}_{i}$ is the estimated partial derivatives design matrix with respect to random effects parameters.

The fit indices of the models were AIC, BIC, and LL. The evaluation criteria of the model also included the mean absolute error (MAE), the mean relative absolute error (RMA), the root mean square error (RMSE), and the coefficient of determination (R^2^).

$$\mathrm{MAE}=\overset{n}{\underset{i=1}{\sum }}\left|\frac{h_{i}‑{\hat{h}}_{i}}{n}\right|$$ (10)

$$\mathrm{RMA}=\frac{\sum_{i=1}^{n}|\frac{h_{i}‑{\hat{h}}_{i}}{{\hat{h}}_{i}}|}{n}\times 100\%$$ (11)

$$RMSE=\sqrt{\frac{\sum_{i=1}^{n}{\left(h_{i}‑{\hat{h}}_{i}\right)}^{2}}{n‑1}}$$ (12)

$$R^{2}=1‑\frac{\sum_{i=1}^{n}{\left(h_{i}‑{\hat{h}}_{i}\right)}^{2}}{\sum_{i=1}^{n}{\left(h_{i}‑{\bar{h}}_{i}\right)}^{2}}$$ (13)

where, h_i_ is the observed value; ${\hat{h}}_{i}$ is the predicted value; $\bar{h_{i}}$ is the mean of the observed values; and n is the sample number.

## Results

### Fixed-effects model

**Fig 2:**
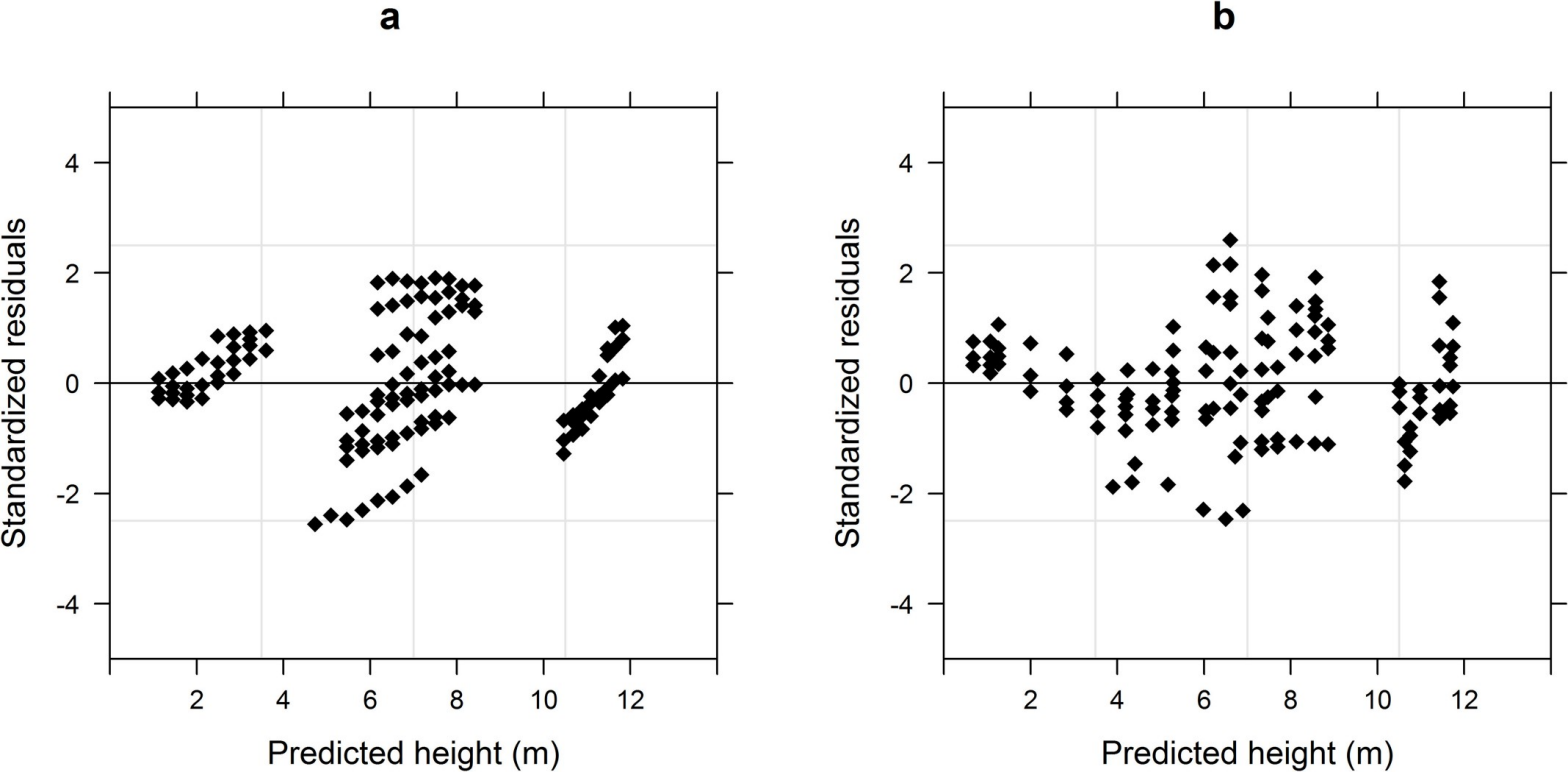
Distributions of standardized residuals. (a) Basic model. (b) Generalized model.

The results of the fixed-effects model (Table 4, see S5 and S6 Tables for model fitting and validation data) indicated that the goodness-of-fit and prediction accuracy of the generalized model were significantly higher than those of the basic model (P < 0.05). However, the accuracies of both models were high (R^2^ ≥ 0.85) and their statistical indices (MAE, RMSE, RMA, and R^2^) were similar. The distribution of the generalized model’s standardized residuals was more uniform than that of the basic model (Fig 2).

**Table 4:** Parameter estimates and variance structure of the three models and the associated fitting and validation statistical criteria.

Project	Parameters	Basic model	Generalized model	Mixed-effects generalized model
Fixed parameters	a_1_	15.732		
	a_2_	0.046	0.114	0.073
	a_3_	1.864	2.917	2.724
	a_4_		−9.049	1.113
	a_5_		1.780	0.317
	a_6_		3.322	−0.318
Variance structure	σ^2^			6.5356E-56
	σi^2^			6.3163E-05
	δ_1_			5.3842e+26
	δ_2_			16.9925
Fitting indices	AIC	341.5642	292.2952	16.5225
	BIC	353.2148	309.7711	42.7364
	LL	−166.7821	−140.1476	0.7388
Testing indices	MAE	0.9938	0.9156	0.2500
	RMA	0.1994	0.1795	0.0476
	RMSE	1.4706	1.1891	0.1225
	R^2^	0.8769	0.9005	0.9897

*δ_1_ and δ_2_ are constant plus power function parameters.

### Mixed-effects model

**Fig 3:**
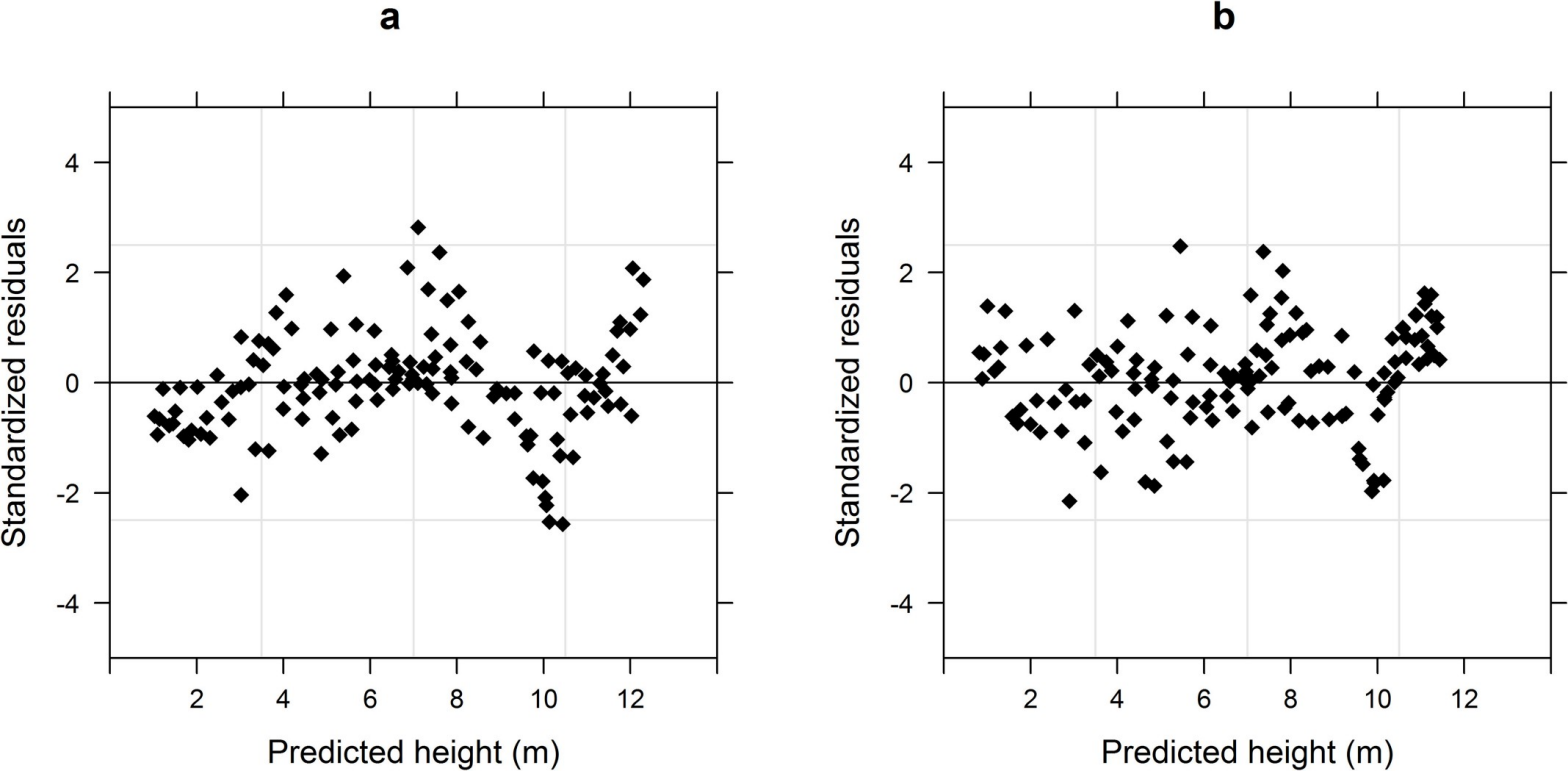
Distributions of standardized residuals of the mixed-effects model. (a) Without constant plus power variance. (b) With variance function.

The mixed-effects model indicated obvious variance heterogeneity (Fig 3A), such that all three variance functions could effectively eliminate the residual heteroscedasticity as the model’s goodness-of-fit increased significantly (P < 0.001) (Table 5). The constant plus power function (Eq (3.3)) yielded maximal changes in AIC, BIC, and LL, and this variance function was finally selected to eliminate heteroscedasticity, which was effective as indicated by the uniform distribution of the model’s standardized residuals after this treatment (Fig 3B).

Among the three models, the mixed-effects model that included climatic factors indicated a better fit and presented the lowest AIC and BIC values as well as the highest LL value (Table 5). In addition, the generalized mixed-effects model also showed the most accurate performance upon validation (Table 5) and improved the prediction performance.

**Table 5:** Mixed-effects model performance with different variance functions.

Model	Variance functions	AIC	BIC	LL	LRT	*P*
Eq (3)	None	58.666	79.055	−22.333		
Eq (3.1)	Power	24.676	47.977	−4.338	35.990	< 0.0001
Eq (3.2)	Exponential	21.235	44.537	−2.618	39.431	< 0.0001
Eq (3.3)	Constant plus power	16.522	42.737	0.739	46.144	< 0.0001

### Effects of climate variables on the height growth of mongolian pine

**Fig 4:**
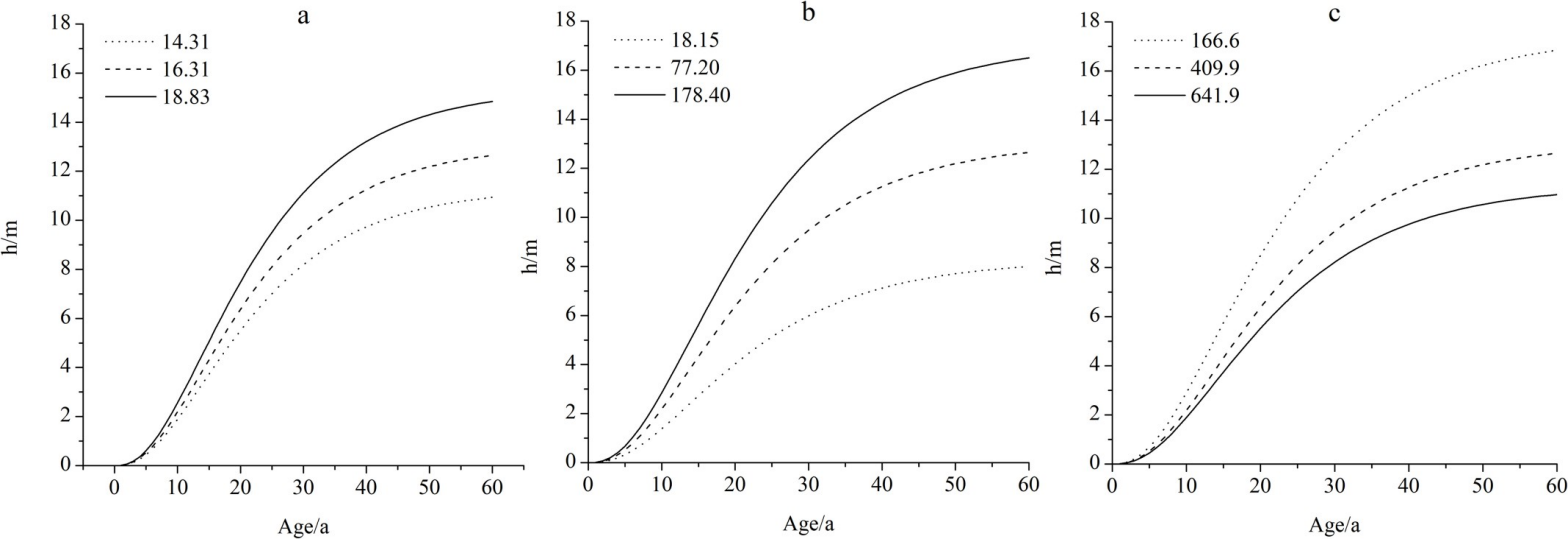
Effects of MTM, PNP, and PGP on tree height growth of mongolian pine. Subplot a to c represent changes in tree height growth with MTM, PNP, and PGP, respectively. (a) MTM = 14.31°C, 16.31°C, and 18.83°C; PNP = 77.2 mm; PGP = 409.9 mm; (b) PNP = 18.15 mm, 77.2 mm, and 178.4 mm; MTM = 16.31°C; PGP = 409.9 mm; (c) PGP = 166.6 mm, 409.9 mm, and 641.9 mm; MTM = 16.31°C; PNP = 77.2 mm.

To determine the effects of MTM, PNP, and PGP on the dominant height growth, two climatic factors were fixed to obtain the relationship between the height growth and the other remaining climatic factors (Fig 4). In the simulation, climatic factors (except the target climatic factors) were replaced by the means from 1974 to 2015 and the target variables were taken as the maximum, average, and minimum. These factors directly affect the estimation of maximum tree height. The effect of MTM and PNP was positive while that of PGP was negative indicating that the tree height increased with increasing MTM and PNP (Fig 4A and 4B) and decreased with increasing PGP (Fig 4C). Therefore, different from the basic model, the model with climatic factors could dynamically predict the tree height growth for one or more years.

## Discussion

Based on the biological Chapman-Richards model, a climate-sensitive dominant height growth model for Mongolian pine was established. Temperature and precipitation are the limiting factors for tree growth [33,34]. MTM, PNP, and PGP significantly affected the height growth of Mongolian pine in Zhanggutai and the three climatic factors all had significant effects on the asymptotic parameters of the Chapman-Richards model. MTM and PNP were positively correlated with tree height, while PGP was negatively correlated. Using the dominant tree height model with climatic factors clearly improved the performance of the model.

May is a period of vigorous height growth for Mongolian pine [35]. The mean temperature in May has a positive effect on the height growth of Mongolian pine; this is consistent with Messaoud and Chen's finding that temperature-related variables positively correlated with tree height [11]. Temperature often affects the growth time [36] and growth rate of tree height, as low temperatures can impede the division and specialization of cambium and meristem cells [37], whereas warmer temperatures promote the activity of photosynthetic enzymes and improve the efficiency of photosynthesis, allowing the accumulation of more nutrients and carbohydrates for distribution to the trunk [38]. Furthermore, warmer temperatures release restrictions placed by low temperatures on water and nutrient uptake in roots [39], promoting soil animal and fungoid activity while improving root water absorption and nutrient exchange, which encourages the earlier arrival of active height growth [33]. In addition, the mean temperature in May was significantly correlated with growing degree-days above 5°C (Table 2), reflecting the amount of heat resources that plants could obtain. A higher temperature in the growing season always means more material accumulation in Mongolian pine [25]. Similar results were found in studies of Mongolian pine plantations in the Mu Us sandy land and the Naiman Region of the Horqin sandy land [25,40].

Tree height is also very sensitive to water [41], and its growth is often associated with precipitation in both current and previous years because precipitation has a lagged effect on growth [14]. It was found that the precipitation from the previous October to the current April in Zhanggutai significantly promoted the height growth of Mongolian pine (Fig 4B). The growth of Mongolian pine plantations is influenced by both precipitation and groundwater [42]. The height growth period ranges from late April to early June [35]. Given that precipitation in May only accounts for approximately 8% of the annual precipitation (1974–2015) in Zhanggutai (even none in some years such as 1986). So the water supply from the previous October to the current April is critical to the height growth of Mongolian pine. In Zhanggutai, the forest soil generally thaws completely by the end of April [42], which happens to be the prophase of the height growth. Meanwhile, the increasing precipitation in November, which increases the soil water content in winter benefits both fine root dynamics and water absorption in spring [43,44]. Therefore, the higher the precipitation from October to April, the more water can be provided, and the greater the height growth of Mongolian pine.

From these results, we can infer that climatic factors in the current year, except for mean temperature in May, had little effect on the height growth of Mongolian pine. However, many factors in the previous year have a significant influence on height growth. As Mongolian pine is a pre-growing tree species, height growth depends on the growth of the top bud in the previous year and the accumulation of nutrients in the trees [45]. We suggest that negative responses of tree height growth to precipitation during the previous year’s growing season are due to decreasing light intensity and temperature caused by increasing temperature, resulting in fewer nutrients being produced by photosynthesis. Meanwhile, sufficient precipitation prolongs the growth season of trees and increases the consumption of nutrients for tree growth [46], such that fewer nutrients are available for buds, resulting in decreased height growth in the following year.

Although the model presented has high reliability and accuracy based on the goodness-of-fit and predictive accuracy values, tree height growth is also influenced by site quality, stand density, silvicultural measure, and other factors [47]. In this study, we only analyzed the effects of climate variables on height growth and incorporated random parameters to reflect differences in tree height growth among different plots.

## Conclusions

This study quantified the effects of climate factors on the height growth of Mongolian pine, of which mean temperature in May (MTM), precipitation from October to April (PNP), and precipitation in the previous growing season (PGP) were found to be the most influential. Tree height was positively correlated with MTM and PNP and negatively correlated with PGP. Incorporating random parameters into the model improved the model performance and reflected differences between different sites. Therefore, our model can be used to predict the height growth of Mongolian pine under changing climate.

## Supporting information

S1 Table Values of aggregate data shown in Table 1. (DOCX)

S2 Table Values of fitting data shown in Table 1. (DOCX)

S3 Table Values of validation data shown in Table 1. (DOCX)

S4 Table Origin meteorological observations shown in Table 2 and Table 3. (DOCX)

S5 Table Values for calculating testing indices shown in Table 4. (DOCX)

S6 Table Values for model fitting and parameters estimate shown in Table 4. (DOCX)
