# Supplementary material for: Impact of climate factors on height growth of Pinus sylvestris var. mongolica
Source: PLoS One. 2019 Mar 11;14(3):e0213509. doi: 10.1371/journal.pone.0213509 (PMC6411114; doi:10.1371/journal.pone.0213509)
Supplement: S1 Table — (DOCX) [file pone.0213509.s001.docx]

**Supporting Information to:**

**Impact of climate factors on height growth of *Pinus sylvestris* var. *mongolica***

Yanping Zhou, Zeyong Lei, Fengyan Zhou, Yangang Han, Deliang Yu, Yansong Zhang

**S1 Table. Values of aggregate data shown in Table 1.**

| age | h | H | D | CW |
| --- | --- | --- | --- | --- |
| 13 | 4.24 | 4.2 | 10.92 | 2.83 |
| 12 | 3.88 |  |  |  |
| 11 | 3.63 |  |  |  |
| 10 | 3.4 |  |  |  |
| 9 | 2.8 |  |  |  |
| 8 | 2.18 |  |  |  |
| 7 | 1.75 |  |  |  |
| 6 | 1.38 |  |  |  |
| 13 | 4.4 | 4.4 | 11.55 | 3.75 |
| 12 | 3.9 |  |  |  |
| 11 | 3.45 |  |  |  |
| 10 | 2.85 |  |  |  |
| 9 | 2.27 |  |  |  |
| 8 | 1.8 |  |  |  |
| 7 | 1.36 |  |  |  |
| 6 | 0.95 |  |  |  |
| 13 | 4.45 | 4.5 | 10.82 | 3.45 |
| 12 | 4.05 |  |  |  |
| 11 | 3.7 |  |  |  |
| 10 | 3.25 |  |  |  |
| 9 | 2.6 |  |  |  |
| 8 | 2 |  |  |  |
| 7 | 1.53 |  |  |  |
| 6 | 1.15 |  |  |  |
| 13 | 4.7 | 4.7 | 12.96 | 3.65 |
| 12 | 4.15 |  |  |  |
| 11 | 3.7 |  |  |  |
| 10 | 3.1 |  |  |  |
| 9 | 2.5 |  |  |  |
| 8 | 2.05 |  |  |  |
| 7 | 1.6 |  |  |  |
| 6 | 1.2 |  |  |  |
| 13 | 4.64 | 4.6 | 9.55 | 3.25 |
| 12 | 4.3 |  |  |  |
| 11 | 3.73 |  |  |  |
| 10 | 2.87 |  |  |  |
| 9 | 2.3 |  |  |  |
| 8 | 1.7 |  |  |  |
| 7 | 1.2 |  |  |  |
| 6 | 0.88 |  |  |  |
| 13 | 3.85 | 3.9 | 9.55 | 3.32 |
| 12 | 3.35 |  |  |  |
| 11 | 2.9 |  |  |  |
| 10 | 2.6 |  |  |  |
| 9 | 1.75 |  |  |  |
| 8 | 1.6 |  |  |  |
| 7 | 1.3 |  |  |  |
| 6 | 1.02 |  |  |  |
| 13 | 4.58 | 4.6 | 9.9 | 3.49 |
| 12 | 4 |  |  |  |
| 11 | 3.6 |  |  |  |
| 10 | 2.91 |  |  |  |
| 9 | 2.15 |  |  |  |
| 8 | 1.93 |  |  |  |
| 7 | 1.65 |  |  |  |
| 6 | 1.33 |  |  |  |
| 13 | 4.35 | 4.4 | 10.03 | 3.64 |
| 12 | 3.93 |  |  |  |
| 11 | 3.4 |  |  |  |
| 10 | 2.8 |  |  |  |
| 9 | 2.15 |  |  |  |
| 8 | 1.7 |  |  |  |
| 7 | 1.45 |  |  |  |
| 6 | 1.1 |  |  |  |
| 13 | 4.26 | 4.3 | 11.9 | 3.12 |
| 12 | 3.5 |  |  |  |
| 11 | 2.93 |  |  |  |
| 10 | 2.63 |  |  |  |
| 9 | 2.06 |  |  |  |
| 8 | 1.65 |  |  |  |
| 7 | 1.3 |  |  |  |
| 6 | 1.06 |  |  |  |
| 13 | 4.2 | 4.2 | 9.87 | 3.33 |
| 12 | 3.6 |  |  |  |
| 11 | 3.17 |  |  |  |
| 10 | 2.5 |  |  |  |
| 9 | 1.9 |  |  |  |
| 8 | 1.6 |  |  |  |
| 7 | 1.3 |  |  |  |
| 6 | 1 |  |  |  |
| 13 | 4.3 | 4.3 | 10.06 | 2.88 |
| 12 | 3.75 |  |  |  |
| 11 | 3.1 |  |  |  |
| 10 | 2.55 |  |  |  |
| 9 | 1.9 |  |  |  |
| 8 | 1.57 |  |  |  |
| 7 | 1.25 |  |  |  |
| 6 | 0.95 |  |  |  |
| 13 | 3.8 | 3.8 | 11.01 | 3.41 |
| 12 | 3.45 |  |  |  |
| 11 | 2.9 |  |  |  |
| 10 | 2.15 |  |  |  |
| 9 | 1.65 |  |  |  |
| 8 | 1.3 |  |  |  |
| 7 | 0.8 |  |  |  |
| 6 | 0.6 |  |  |  |
| 13 | 4.7 | 4.7 | 10.66 | 3.35 |
| 12 | 4.1 |  |  |  |
| 11 | 3.5 |  |  |  |
| 10 | 2.8 |  |  |  |
| 9 | 2.1 |  |  |  |
| 8 | 1.6 |  |  |  |
| 7 | 1.3 |  |  |  |
| 6 | 1 |  |  |  |
| 13 | 4.9 | 4.9 | 11.46 | 3.99 |
| 12 | 4.3 |  |  |  |
| 11 | 3.6 |  |  |  |
| 10 | 2.8 |  |  |  |
| 9 | 2.1 |  |  |  |
| 8 | 1.7 |  |  |  |
| 7 | 1.4 |  |  |  |
| 6 | 0.9 |  |  |  |
| 13 | 4.1 | 4.1 | 10.7 | 3.28 |
| 12 | 3.6 |  |  |  |
| 11 | 3.1 |  |  |  |
| 10 | 2.5 |  |  |  |
| 9 | 2.1 |  |  |  |
| 8 | 1.6 |  |  |  |
| 7 | 1.3 |  |  |  |
| 6 | 0.9 |  |  |  |
| 13 | 3.7 | 3.7 | 9.87 | 3.39 |
| 12 | 3.3 |  |  |  |
| 11 | 2.7 |  |  |  |
| 10 | 2.3 |  |  |  |
| 9 | 1.9 |  |  |  |
| 8 | 1.3 |  |  |  |
| 7 | 1 |  |  |  |
| 6 | 0.7 |  |  |  |
| 13 | 3.6 | 3.6 | 10.12 | 2.78 |
| 12 | 3.3 |  |  |  |
| 11 | 2.8 |  |  |  |
| 10 | 2.3 |  |  |  |
| 9 | 2.1 |  |  |  |
| 8 | 1.6 |  |  |  |
| 7 | 1.1 |  |  |  |
| 6 | 0.9 |  |  |  |
| 13 | 3.2 | 3.2 | 10.38 | 2.53 |
| 12 | 2.9 |  |  |  |
| 11 | 2.4 |  |  |  |
| 10 | 2.1 |  |  |  |
| 9 | 1.6 |  |  |  |
| 8 | 1.3 |  |  |  |
| 7 | 1 |  |  |  |
| 6 | 0.4 |  |  |  |
| 13 | 2.7 | 2.7 | 15.85 | 2 |
| 12 | 2.2 |  |  |  |
| 11 | 1.7 |  |  |  |
| 10 | 1.3 |  |  |  |
| 9 | 0.9 |  |  |  |
| 8 | 0.7 |  |  |  |
| 7 | 0.4 |  |  |  |
| 6 | 0.3 |  |  |  |
| 13 | 3.4 | 3.4 | 9.49 | 2.58 |
| 12 | 2.9 |  |  |  |
| 11 | 2.4 |  |  |  |
| 10 | 1.9 |  |  |  |
| 9 | 1.4 |  |  |  |
| 8 | 1 |  |  |  |
| 7 | 0.7 |  |  |  |
| 6 | 0.5 |  |  |  |
| 23 | 6.03 | 6 | 20.82 | 6.5 |
| 22 | 5.52 |  |  |  |
| 21 | 5.3 |  |  |  |
| 20 | 4.8 |  |  |  |
| 19 | 4.35 |  |  |  |
| 18 | 3.87 |  |  |  |
| 17 | 3.45 |  |  |  |
| 16 | 3 |  |  |  |
| 23 | 5.9 | 5.9 | 16.87 | 4.71 |
| 22 | 5.3 |  |  |  |
| 21 | 4.9 |  |  |  |
| 20 | 4.4 |  |  |  |
| 19 | 4 |  |  |  |
| 18 | 3.4 |  |  |  |
| 17 | 3.1 |  |  |  |
| 16 | 2.7 |  |  |  |
| 23 | 5.7 | 5.7 | 17.63 | 4.46 |
| 22 | 5.15 |  |  |  |
| 21 | 4.6 |  |  |  |
| 20 | 4.21 |  |  |  |
| 19 | 3.91 |  |  |  |
| 18 | 3.42 |  |  |  |
| 17 | 3.05 |  |  |  |
| 16 | 2.55 |  |  |  |
| 23 | 5.52 | 5.5 | 20.12 | 3.85 |
| 22 | 5.06 |  |  |  |
| 21 | 4.53 |  |  |  |
| 20 | 4.01 |  |  |  |
| 19 | 3.47 |  |  |  |
| 18 | 3.07 |  |  |  |
| 17 | 2.7 |  |  |  |
| 16 | 2.22 |  |  |  |
| 25 | 7.7 | 7.7 | 17.25 | 3.53 |
| 24 | 7.5 |  |  |  |
| 23 | 7.3 |  |  |  |
| 22 | 6.9 |  |  |  |
| 21 | 6.4 |  |  |  |
| 20 | 6.2 |  |  |  |
| 19 | 5.9 |  |  |  |
| 18 | 5.6 |  |  |  |
| 25 | 6.8 | 6.8 | 15.66 | 4.67 |
| 24 | 6.5 |  |  |  |
| 23 | 6.2 |  |  |  |
| 22 | 5.8 |  |  |  |
| 21 | 5.4 |  |  |  |
| 20 | 5 |  |  |  |
| 19 | 4.8 |  |  |  |
| 18 | 4.4 |  |  |  |
| 25 | 7.4 | 7.4 | 16.49 | 4.82 |
| 24 | 6.9 |  |  |  |
| 23 | 6.5 |  |  |  |
| 22 | 5.9 |  |  |  |
| 21 | 5.6 |  |  |  |
| 20 | 5.2 |  |  |  |
| 19 | 4.8 |  |  |  |
| 18 | 4.5 |  |  |  |
| 25 | 7.4 | 7.4 | 16.77 | 3.6 |
| 24 | 6.9 |  |  |  |
| 23 | 6.2 |  |  |  |
| 22 | 5.7 |  |  |  |
| 21 | 5.1 |  |  |  |
| 20 | 4.6 |  |  |  |
| 19 | 4 |  |  |  |
| 18 | 3.6 |  |  |  |
| 25 | 9.2 | 9.2 | 16.87 | 3.7 |
| 24 | 8.8 |  |  |  |
| 23 | 8.3 |  |  |  |
| 22 | 7.7 |  |  |  |
| 21 | 7.2 |  |  |  |
| 20 | 6.6 |  |  |  |
| 19 | 6.1 |  |  |  |
| 18 | 5.6 |  |  |  |
| 25 | 8.4 | 8.4 | 16.17 | 3.33 |
| 24 | 8.2 |  |  |  |
| 23 | 7.7 |  |  |  |
| 22 | 7.3 |  |  |  |
| 21 | 6.9 |  |  |  |
| 20 | 6.4 |  |  |  |
| 19 | 5.8 |  |  |  |
| 18 | 5.2 |  |  |  |
| 25 | 7.6 | 7.6 | 17.51 | 4.03 |
| 24 | 7.1 |  |  |  |
| 23 | 6.7 |  |  |  |
| 22 | 6.3 |  |  |  |
| 21 | 6 |  |  |  |
| 20 | 5.6 |  |  |  |
| 19 | 5.1 |  |  |  |
| 18 | 4.8 |  |  |  |
| 25 | 8 | 8 | 17.19 | 5.08 |
| 24 | 7.6 |  |  |  |
| 23 | 7.1 |  |  |  |
| 22 | 6.5 |  |  |  |
| 21 | 6 |  |  |  |
| 20 | 5.3 |  |  |  |
| 19 | 4.7 |  |  |  |
| 18 | 4.4 |  |  |  |
| 25 | 8.1 | 8.1 | 21.33 | 5.66 |
| 24 | 7.7 |  |  |  |
| 23 | 7.1 |  |  |  |
| 22 | 6.7 |  |  |  |
| 21 | 6.2 |  |  |  |
| 20 | 5.6 |  |  |  |
| 19 | 5.4 |  |  |  |
| 18 | 5.1 |  |  |  |
| 25 | 8 | 8 | 18.97 | 4.38 |
| 24 | 7.7 |  |  |  |
| 23 | 7.4 |  |  |  |
| 22 | 7.2 |  |  |  |
| 21 | 6.8 |  |  |  |
| 20 | 6.2 |  |  |  |
| 19 | 5.6 |  |  |  |
| 18 | 5.1 |  |  |  |
| 25 | 7.2 | 7.2 | 18.68 | 4.83 |
| 24 | 6.8 |  |  |  |
| 23 | 6.3 |  |  |  |
| 22 | 5.7 |  |  |  |
| 21 | 5.2 |  |  |  |
| 20 | 4.6 |  |  |  |
| 19 | 4 |  |  |  |
| 18 | 3.4 |  |  |  |
| 25 | 8.6 | 8.6 | 18.24 | 4.18 |
| 24 | 8 |  |  |  |
| 23 | 7.5 |  |  |  |
| 22 | 7.3 |  |  |  |
| 21 | 6.6 |  |  |  |
| 20 | 6.2 |  |  |  |
| 19 | 5.4 |  |  |  |
| 18 | 4.8 |  |  |  |
| 25 | 6.7 | 6.7 | 15.41 | 4.08 |
| 24 | 6.2 |  |  |  |
| 23 | 5.8 |  |  |  |
| 22 | 5.4 |  |  |  |
| 21 | 5.2 |  |  |  |
| 20 | 5 |  |  |  |
| 19 | 4.6 |  |  |  |
| 18 | 4.1 |  |  |  |
| 25 | 8.2 | 8.2 | 15.22 | 3.45 |
| 24 | 7.7 |  |  |  |
| 23 | 7.2 |  |  |  |
| 22 | 6.7 |  |  |  |
| 21 | 6.1 |  |  |  |
| 20 | 5.5 |  |  |  |
| 19 | 5 |  |  |  |
| 18 | 4.2 |  |  |  |
| 25 | 7.6 | 7.6 | 16.07 | 3.89 |
| 24 | 7.4 |  |  |  |
| 23 | 7.2 |  |  |  |
| 22 | 6.9 |  |  |  |
| 21 | 6.5 |  |  |  |
| 20 | 5.9 |  |  |  |
| 19 | 5.6 |  |  |  |
| 18 | 5.1 |  |  |  |
| 25 | 6.7 | 6.7 | 16.23 | 4.26 |
| 24 | 6.3 |  |  |  |
| 23 | 5.9 |  |  |  |
| 22 | 5.4 |  |  |  |
| 21 | 5 |  |  |  |
| 20 | 4.5 |  |  |  |
| 19 | 4.1 |  |  |  |
| 18 | 3.7 |  |  |  |
| 23 | 5.18 | 5.2 | 19.1 | 4.47 |
| 22 | 4.85 |  |  |  |
| 21 | 4.42 |  |  |  |
| 20 | 3.88 |  |  |  |
| 19 | 3.34 |  |  |  |
| 18 | 3 |  |  |  |
| 17 | 2.76 |  |  |  |
| 16 | 2.35 |  |  |  |
| 23 | 5.15 | 5.2 | 16.3 | 3.95 |
| 22 | 4.52 |  |  |  |
| 21 | 4.03 |  |  |  |
| 20 | 3.52 |  |  |  |
| 19 | 3.16 |  |  |  |
| 18 | 2.83 |  |  |  |
| 17 | 2.43 |  |  |  |
| 16 | 2.04 |  |  |  |
| 23 | 5.96 | 6 | 17.7 | 3.86 |
| 22 | 5.66 |  |  |  |
| 21 | 5.03 |  |  |  |
| 20 | 4.4 |  |  |  |
| 19 | 3.81 |  |  |  |
| 18 | 3.3 |  |  |  |
| 17 | 2.99 |  |  |  |
| 16 | 2.5 |  |  |  |
| 23 | 5.39 | 5.4 | 17.44 | 4.69 |
| 22 | 5.07 |  |  |  |
| 21 | 4.48 |  |  |  |
| 20 | 4.08 |  |  |  |
| 19 | 3.61 |  |  |  |
| 18 | 3.2 |  |  |  |
| 17 | 2.7 |  |  |  |
| 16 | 2.35 |  |  |  |
| 27 | 9.9 | 9.9 | 19.39 | 3.97 |
| 26 | 9.7 |  |  |  |
| 25 | 9.3 |  |  |  |
| 24 | 9 |  |  |  |
| 23 | 8.8 |  |  |  |
| 22 | 8.5 |  |  |  |
| 21 | 8.1 |  |  |  |
| 20 | 7.8 |  |  |  |
| 27 | 10.3 | 10.3 | 19.42 | 4.35 |
| 26 | 9.9 |  |  |  |
| 25 | 9.7 |  |  |  |
| 24 | 9.3 |  |  |  |
| 23 | 8.8 |  |  |  |
| 22 | 8.4 |  |  |  |
| 21 | 8.1 |  |  |  |
| 20 | 7.6 |  |  |  |
| 27 | 9.6 | 9.6 | 18.84 | 4.95 |
| 26 | 9.3 |  |  |  |
| 25 | 9 |  |  |  |
| 24 | 8.8 |  |  |  |
| 23 | 8.4 |  |  |  |
| 22 | 8 |  |  |  |
| 21 | 7.7 |  |  |  |
| 20 | 7.3 |  |  |  |
| 27 | 9.9 | 9.9 | 19.1 | 3.8 |
| 26 | 9.5 |  |  |  |
| 25 | 9.4 |  |  |  |
| 24 | 9.1 |  |  |  |
| 23 | 8.9 |  |  |  |
| 22 | 8.7 |  |  |  |
| 21 | 8.4 |  |  |  |
| 20 | 8.1 |  |  |  |
| 27 | 8.5 | 8.5 | 18.72 | 3.79 |
| 26 | 8.2 |  |  |  |
| 25 | 7.8 |  |  |  |
| 24 | 7.3 |  |  |  |
| 23 | 6.8 |  |  |  |
| 22 | 6.3 |  |  |  |
| 21 | 5.8 |  |  |  |
| 20 | 5.4 |  |  |  |
| 27 | 8.1 | 8.1 | 19.29 | 3.95 |
| 26 | 7.8 |  |  |  |
| 25 | 7.4 |  |  |  |
| 24 | 7 |  |  |  |
| 23 | 6.5 |  |  |  |
| 22 | 6.1 |  |  |  |
| 21 | 5.8 |  |  |  |
| 20 | 5.3 |  |  |  |
| 27 | 8.4 | 8.4 | 19.77 | 5.2 |
| 26 | 8.2 |  |  |  |
| 25 | 8 |  |  |  |
| 24 | 7.5 |  |  |  |
| 23 | 7.1 |  |  |  |
| 22 | 6.7 |  |  |  |
| 21 | 6.3 |  |  |  |
| 20 | 6 |  |  |  |
| 27 | 8.6 | 8.6 | 19.99 | 4.72 |
| 26 | 8.3 |  |  |  |
| 25 | 8.1 |  |  |  |
| 24 | 7.8 |  |  |  |
| 23 | 7.5 |  |  |  |
| 22 | 7.4 |  |  |  |
| 21 | 7.1 |  |  |  |
| 20 | 6.8 |  |  |  |
| 27 | 10.1 | 10.1 | 19.1 | 2.91 |
| 26 | 9.7 |  |  |  |
| 25 | 9.2 |  |  |  |
| 24 | 8.7 |  |  |  |
| 23 | 8 |  |  |  |
| 22 | 7.7 |  |  |  |
| 21 | 6.9 |  |  |  |
| 20 | 6.6 |  |  |  |
| 27 | 9.2 | 9.2 | 19.1 | 4.34 |
| 26 | 9.1 |  |  |  |
| 25 | 8.7 |  |  |  |
| 24 | 8.5 |  |  |  |
| 23 | 8 |  |  |  |
| 22 | 7.6 |  |  |  |
| 21 | 7 |  |  |  |
| 20 | 6.5 |  |  |  |
| 27 | 8.7 | 8.7 | 21.26 | 4 |
| 26 | 8.3 |  |  |  |
| 25 | 8.1 |  |  |  |
| 24 | 7.7 |  |  |  |
| 23 | 7.2 |  |  |  |
| 22 | 6.9 |  |  |  |
| 21 | 6.4 |  |  |  |
| 20 | 6 |  |  |  |
| 27 | 10.1 | 10.1 | 20.85 | 5.42 |
| 26 | 9.9 |  |  |  |
| 25 | 9.6 |  |  |  |
| 24 | 9 |  |  |  |
| 23 | 8.5 |  |  |  |
| 22 | 8.1 |  |  |  |
| 21 | 7.6 |  |  |  |
| 20 | 7.1 |  |  |  |
| 27 | 9 | 9 | 19.64 | 4.84 |
| 26 | 8.8 |  |  |  |
| 25 | 8.7 |  |  |  |
| 24 | 8.5 |  |  |  |
| 23 | 8.1 |  |  |  |
| 22 | 7.8 |  |  |  |
| 21 | 7.3 |  |  |  |
| 20 | 6.8 |  |  |  |
| 27 | 11.1 | 11.1 | 21.33 | 4.13 |
| 26 | 10.8 |  |  |  |
| 25 | 10.6 |  |  |  |
| 24 | 10 |  |  |  |
| 23 | 9.6 |  |  |  |
| 22 | 8.9 |  |  |  |
| 21 | 8.4 |  |  |  |
| 20 | 8.1 |  |  |  |
| 27 | 9.4 | 9.4 | 20.85 | 5.44 |
| 26 | 9.1 |  |  |  |
| 25 | 8.9 |  |  |  |
| 24 | 8.5 |  |  |  |
| 23 | 8.1 |  |  |  |
| 22 | 7.7 |  |  |  |
| 21 | 7.3 |  |  |  |
| 20 | 6.9 |  |  |  |
| 27 | 9 | 9 | 21.87 | 5.17 |
| 26 | 8.8 |  |  |  |
| 25 | 8.6 |  |  |  |
| 24 | 8.3 |  |  |  |
| 23 | 8.1 |  |  |  |
| 22 | 7.9 |  |  |  |
| 21 | 7.6 |  |  |  |
| 20 | 7.3 |  |  |  |
| 27 | 9.1 | 9.1 | 18.65 | 3.75 |
| 26 | 8.8 |  |  |  |
| 25 | 8.5 |  |  |  |
| 24 | 8.1 |  |  |  |
| 23 | 7.9 |  |  |  |
| 22 | 7.6 |  |  |  |
| 21 | 7 |  |  |  |
| 20 | 6.5 |  |  |  |
| 27 | 7.8 | 7.8 | 18.14 | 3.2 |
| 26 | 7.7 |  |  |  |
| 25 | 7.4 |  |  |  |
| 24 | 7.2 |  |  |  |
| 23 | 7 |  |  |  |
| 22 | 6.8 |  |  |  |
| 21 | 6.6 |  |  |  |
| 20 | 6.4 |  |  |  |
| 27 | 9.2 | 9.2 | 17.48 | 4.73 |
| 26 | 8.9 |  |  |  |
| 25 | 8.6 |  |  |  |
| 24 | 8.4 |  |  |  |
| 23 | 8.2 |  |  |  |
| 22 | 7.9 |  |  |  |
| 21 | 7.7 |  |  |  |
| 20 | 7.4 |  |  |  |
| 27 | 9.1 | 9.1 | 17.63 | 3.5 |
| 26 | 8.8 |  |  |  |
| 25 | 8.3 |  |  |  |
| 24 | 7.8 |  |  |  |
| 23 | 7.5 |  |  |  |
| 22 | 7.3 |  |  |  |
| 21 | 7.2 |  |  |  |
| 20 | 6.8 |  |  |  |
| 42 | 9.9 | 9.9 | 20.94 | 4.79 |
| 41 | 9.7 |  |  |  |
| 40 | 9.5 |  |  |  |
| 39 | 9.4 |  |  |  |
| 38 | 9.2 |  |  |  |
| 37 | 8.9 |  |  |  |
| 36 | 8.7 |  |  |  |
| 35 | 8.5 |  |  |  |
| 42 | 12 | 12 | 22.54 | 5.1 |
| 41 | 11.7 |  |  |  |
| 40 | 11.3 |  |  |  |
| 39 | 10.8 |  |  |  |
| 38 | 10.4 |  |  |  |
| 37 | 10 |  |  |  |
| 36 | 9.6 |  |  |  |
| 35 | 9.1 |  |  |  |
| 42 | 12.8 | 12.8 | 25.15 | 5.3 |
| 41 | 12.6 |  |  |  |
| 40 | 12.4 |  |  |  |
| 39 | 11.9 |  |  |  |
| 38 | 11.4 |  |  |  |
| 37 | 11.1 |  |  |  |
| 36 | 10.7 |  |  |  |
| 35 | 10.4 |  |  |  |
| 42 | 12.8 | 12.8 | 26.99 | 5.29 |
| 41 | 12.7 |  |  |  |
| 40 | 12.5 |  |  |  |
| 39 | 12.4 |  |  |  |
| 38 | 12.2 |  |  |  |
| 37 | 12 |  |  |  |
| 36 | 11.8 |  |  |  |
| 35 | 11.6 |  |  |  |
| 42 | 11.9 | 11.9 | 22.79 | 4.5 |
| 41 | 11.6 |  |  |  |
| 40 | 11.1 |  |  |  |
| 39 | 10.6 |  |  |  |
| 38 | 10.1 |  |  |  |
| 37 | 9.8 |  |  |  |
| 36 | 9.5 |  |  |  |
| 35 | 9.1 |  |  |  |
| 42 | 13.7 | 13.7 | 24.67 | 6.32 |
| 41 | 13.5 |  |  |  |
| 40 | 13 |  |  |  |
| 39 | 12.5 |  |  |  |
| 38 | 12.1 |  |  |  |
| 37 | 11.6 |  |  |  |
| 36 | 11.3 |  |  |  |
| 35 | 10.5 |  |  |  |
| 42 | 13.6 | 13.6 | 25.94 | 6.06 |
| 41 | 13.3 |  |  |  |
| 40 | 12.7 |  |  |  |
| 39 | 12.2 |  |  |  |
| 38 | 11.4 |  |  |  |
| 37 | 10.7 |  |  |  |
| 36 | 10.1 |  |  |  |
| 35 | 9.8 |  |  |  |
| 42 | 11.6 | 11.6 | 22.41 | 4.75 |
| 41 | 11.4 |  |  |  |
| 40 | 11 |  |  |  |
| 39 | 10.4 |  |  |  |
| 38 | 10 |  |  |  |
| 37 | 9.6 |  |  |  |
| 36 | 9.3 |  |  |  |
| 35 | 9.1 |  |  |  |
| 42 | 11.3 | 11.3 | 24.67 | 4.83 |
| 41 | 11.1 |  |  |  |
| 40 | 10.8 |  |  |  |
| 39 | 10.7 |  |  |  |
| 38 | 10.3 |  |  |  |
| 37 | 9.8 |  |  |  |
| 36 | 9.6 |  |  |  |
| 35 | 9.2 |  |  |  |
| 42 | 11.4 | 11.4 | 24.83 | 5.96 |
| 41 | 11 |  |  |  |
| 40 | 10.6 |  |  |  |
| 39 | 10.4 |  |  |  |
| 38 | 9.8 |  |  |  |
| 37 | 9.5 |  |  |  |
| 36 | 9 |  |  |  |
| 35 | 8.6 |  |  |  |
| 42 | 12.3 | 12.3 | 26.36 | 5.49 |
| 41 | 12.2 |  |  |  |
| 40 | 11.8 |  |  |  |
| 39 | 11.5 |  |  |  |
| 38 | 11.2 |  |  |  |
| 37 | 10.9 |  |  |  |
| 36 | 10.7 |  |  |  |
| 35 | 10.4 |  |  |  |
| 42 | 12.6 | 12.6 | 26.1 | 6.36 |
| 41 | 12.3 |  |  |  |
| 40 | 11.9 |  |  |  |
| 39 | 11.5 |  |  |  |
| 38 | 11 |  |  |  |
| 37 | 10.5 |  |  |  |
| 36 | 10.1 |  |  |  |
| 35 | 9.5 |  |  |  |
| 42 | 12 | 12 | 16.93 | 3.13 |
| 41 | 11.8 |  |  |  |
| 40 | 11.5 |  |  |  |
| 39 | 11.3 |  |  |  |
| 38 | 11.1 |  |  |  |
| 37 | 10.7 |  |  |  |
| 36 | 10.3 |  |  |  |
| 35 | 9.8 |  |  |  |
| 42 | 13 | 13 | 18.3 | 3 |
| 41 | 12.6 |  |  |  |
| 40 | 12.2 |  |  |  |
| 39 | 11.8 |  |  |  |
| 38 | 11.4 |  |  |  |
| 37 | 10.9 |  |  |  |
| 36 | 10.7 |  |  |  |
| 35 | 10.4 |  |  |  |
| 42 | 12.7 | 12.7 | 19.29 | 4.23 |
| 41 | 12.4 |  |  |  |
| 40 | 12.1 |  |  |  |
| 39 | 11.4 |  |  |  |
| 38 | 10.6 |  |  |  |
| 37 | 10.1 |  |  |  |
| 36 | 9.7 |  |  |  |
| 35 | 9.5 |  |  |  |
| 42 | 12.3 | 12.3 | 18.97 | 3.82 |
| 41 | 12.1 |  |  |  |
| 40 | 11.7 |  |  |  |
| 39 | 11.1 |  |  |  |
| 38 | 10.6 |  |  |  |
| 37 | 10.1 |  |  |  |
| 36 | 9.5 |  |  |  |
| 35 | 8.8 |  |  |  |
| 42 | 11.1 | 11.1 | 20.37 | 4.29 |
| 41 | 10.8 |  |  |  |
| 40 | 10.5 |  |  |  |
| 39 | 10 |  |  |  |
| 38 | 9.7 |  |  |  |
| 37 | 9.3 |  |  |  |
| 36 | 8.7 |  |  |  |
| 35 | 8.4 |  |  |  |
| 42 | 11.9 | 11.9 | 20.47 | 4.34 |
| 41 | 11.7 |  |  |  |
| 40 | 11.3 |  |  |  |
| 39 | 10.7 |  |  |  |
| 38 | 10.3 |  |  |  |
| 37 | 10 |  |  |  |
| 36 | 9.6 |  |  |  |
| 35 | 9.2 |  |  |  |
| 42 | 12.3 | 12.3 | 19.48 | 4.69 |
| 41 | 11.9 |  |  |  |
| 40 | 11.5 |  |  |  |
| 39 | 10.9 |  |  |  |
| 38 | 10.4 |  |  |  |
| 37 | 9.8 |  |  |  |
| 36 | 9.4 |  |  |  |
| 35 | 9.1 |  |  |  |
| 42 | 11.5 | 11.5 | 21.23 | 3.89 |
| 41 | 11.2 |  |  |  |
| 40 | 10.7 |  |  |  |
| 39 | 10.3 |  |  |  |
| 38 | 9.9 |  |  |  |
| 37 | 9.7 |  |  |  |
| 36 | 9.3 |  |  |  |
| 35 | 8.8 |  |  |  |
