# Supplementary material for: Impact of climate factors on height growth of Pinus sylvestris var. mongolica
Source: PLoS One. 2019 Mar 11;14(3):e0213509. doi: 10.1371/journal.pone.0213509 (PMC6411114; doi:10.1371/journal.pone.0213509)
Supplement: S2 Table — (DOCX) [file pone.0213509.s002.docx]

**Supporting Information to:**

**Impact of climate factors on height growth of *Pinus sylvestris* var. *mongolica***

Yanping Zhou, Zeyong Lei, Fengyan Zhou, Yangang Han, Deliang Yu, Yansong Zhang

**S2 Table. Values of fitting data shown in Table 1.**

| h | t |  | H | D | CW |
| --- | --- | --- | --- | --- | --- |
| 4.24 | 13 | 2.825 | 4.2 | 10.92 | 2.83 |
| 3.88 | 12 |  |  |  |  |
| 3.63 | 11 |  |  |  |  |
| 3.4 | 10 |  |  |  |  |
| 2.8 | 9 |  |  |  |  |
| 2.18 | 8 |  |  |  |  |
| 1.75 | 7 |  |  |  |  |
| 1.38 | 6 |  |  |  |  |
| 4.4 | 13 | 3.75 | 4.4 | 11.55 | 3.75 |
| 3.9 | 12 |  |  |  |  |
| 3.45 | 11 |  |  |  |  |
| 2.85 | 10 |  |  |  |  |
| 2.27 | 9 |  |  |  |  |
| 1.8 | 8 |  |  |  |  |
| 1.36 | 7 |  |  |  |  |
| 0.95 | 6 |  |  |  |  |
| 4.45 | 13 | 3.45 | 4.5 | 10.82 | 3.45 |
| 4.05 | 12 |  |  |  |  |
| 3.7 | 11 |  |  |  |  |
| 3.25 | 10 |  |  |  |  |
| 2.6 | 9 |  |  |  |  |
| 2 | 8 |  |  |  |  |
| 1.53 | 7 |  |  |  |  |
| 1.15 | 6 |  |  |  |  |
| 4.7 | 13 | 3.65 | 4.7 | 12.96 | 3.65 |
| 4.15 | 12 |  |  |  |  |
| 3.7 | 11 |  |  |  |  |
| 3.1 | 10 |  |  |  |  |
| 2.5 | 9 |  |  |  |  |
| 2.05 | 8 |  |  |  |  |
| 1.6 | 7 |  |  |  |  |
| 1.2 | 6 |  |  |  |  |
| 4.64 | 13 | 3.25 | 4.6 | 9.55 | 3.25 |
| 4.3 | 12 |  |  |  |  |
| 3.73 | 11 |  |  |  |  |
| 2.87 | 10 |  |  |  |  |
| 2.3 | 9 |  |  |  |  |
| 1.7 | 8 |  |  |  |  |
| 1.2 | 7 |  |  |  |  |
| 0.88 | 6 |  |  |  |  |
| 3.85 | 13 | 3.315 | 3.9 | 9.55 | 3.32 |
| 3.35 | 12 |  |  |  |  |
| 2.9 | 11 |  |  |  |  |
| 2.6 | 10 |  |  |  |  |
| 1.75 | 9 |  |  |  |  |
| 1.6 | 8 |  |  |  |  |
| 1.3 | 7 |  |  |  |  |
| 1.02 | 6 |  |  |  |  |
| 4.58 | 13 | 3.485 | 4.6 | 9.9 | 3.49 |
| 4 | 12 |  |  |  |  |
| 3.6 | 11 |  |  |  |  |
| 2.91 | 10 |  |  |  |  |
| 2.15 | 9 |  |  |  |  |
| 1.93 | 8 |  |  |  |  |
| 1.65 | 7 |  |  |  |  |
| 1 | 6 |  |  |  |  |
| 4.35 | 13 | 3.635 | 4.4 | 10.03 | 3.64 |
| 3.93 | 12 |  |  |  |  |
| 3.4 | 11 |  |  |  |  |
| 2.8 | 10 |  |  |  |  |
| 2.15 | 9 |  |  |  |  |
| 1.7 | 8 |  |  |  |  |
| 1.45 | 7 |  |  |  |  |
| 1.1 | 6 |  |  |  |  |
| 4.26 | 13 | 3.115 | 4.3 | 11.9 | 3.12 |
| 3.5 | 12 |  |  |  |  |
| 2.93 | 11 |  |  |  |  |
| 2.63 | 10 |  |  |  |  |
| 2.06 | 9 |  |  |  |  |
| 1.65 | 8 |  |  |  |  |
| 1.3 | 7 |  |  |  |  |
| 1.06 | 6 |  |  |  |  |
| 4.2 | 13 | 3.33 | 4.2 | 9.87 | 3.33 |
| 3.6 | 12 |  |  |  |  |
| 3.17 | 11 |  |  |  |  |
| 2.5 | 10 |  |  |  |  |
| 1.9 | 9 |  |  |  |  |
| 1.6 | 8 |  |  |  |  |
| 1.3 | 7 |  |  |  |  |
| 1 | 6 |  |  |  |  |
| 4.3 | 13 | 2.875 | 4.3 | 10.06 | 2.88 |
| 3.75 | 12 |  |  |  |  |
| 3.1 | 11 |  |  |  |  |
| 2.55 | 10 |  |  |  |  |
| 1.9 | 9 |  |  |  |  |
| 1.57 | 8 |  |  |  |  |
| 1.25 | 7 |  |  |  |  |
| 0.95 | 6 |  |  |  |  |
| 3.8 | 13 | 3.41 | 3.8 | 11.01 | 3.41 |
| 3.45 | 12 |  |  |  |  |
| 2.9 | 11 |  |  |  |  |
| 2.15 | 10 |  |  |  |  |
| 1.65 | 9 |  |  |  |  |
| 1.3 | 8 |  |  |  |  |
| 0.8 | 7 |  |  |  |  |
| 0.6 | 6 |  |  |  |  |
| 4.7 | 13 | 3.345 | 4.7 | 10.66 | 3.35 |
| 4.1 | 12 |  |  |  |  |
| 3.5 | 11 |  |  |  |  |
| 2.8 | 10 |  |  |  |  |
| 2.1 | 9 |  |  |  |  |
| 1.6 | 8 |  |  |  |  |
| 1.3 | 7 |  |  |  |  |
| 1 | 6 |  |  |  |  |
| 4.9 | 13 | 3.99 | 4.9 | 11.46 | 3.99 |
| 4.3 | 12 |  |  |  |  |
| 3.6 | 11 |  |  |  |  |
| 2.8 | 10 |  |  |  |  |
| 2.1 | 9 |  |  |  |  |
| 1.7 | 8 |  |  |  |  |
| 1.4 | 7 |  |  |  |  |
| 0.9 | 6 |  |  |  |  |
| 4.1 | 13 | 3.275 | 4.1 | 10.7 | 3.28 |
| 3.6 | 12 |  |  |  |  |
| 3.1 | 11 |  |  |  |  |
| 2.5 | 10 |  |  |  |  |
| 2.1 | 9 |  |  |  |  |
| 1.6 | 8 |  |  |  |  |
| 1.3 | 7 |  |  |  |  |
| 0.9 | 6 |  |  |  |  |
| 3.7 | 13 | 3.39 | 3.7 | 9.87 | 3.39 |
| 3.3 | 12 |  |  |  |  |
| 2.7 | 11 |  |  |  |  |
| 2.3 | 10 |  |  |  |  |
| 1.9 | 9 |  |  |  |  |
| 1.3 | 8 |  |  |  |  |
| 1 | 7 |  |  |  |  |
| 0.7 | 6 |  |  |  |  |
| 6.03 | 23 | 6.495 | 6 | 20.82 | 6.5 |
| 5.52 | 22 |  |  |  |  |
| 5.3 | 21 |  |  |  |  |
| 4.8 | 20 |  |  |  |  |
| 4.35 | 19 |  |  |  |  |
| 3.87 | 18 |  |  |  |  |
| 3.45 | 17 |  |  |  |  |
| 3 | 16 |  |  |  |  |
| 5.9 | 23 | 4.705 | 5.9 | 16.87 | 4.71 |
| 5.3 | 22 |  |  |  |  |
| 4.9 | 21 |  |  |  |  |
| 4.4 | 20 |  |  |  |  |
| 4 | 19 |  |  |  |  |
| 3.4 | 18 |  |  |  |  |
| 3.1 | 17 |  |  |  |  |
| 2.7 | 16 |  |  |  |  |
| 5.7 | 23 | 4.455 | 5.7 | 17.63 | 4.46 |
| 5.15 | 22 |  |  |  |  |
| 4.6 | 21 |  |  |  |  |
| 4.21 | 20 |  |  |  |  |
| 3.91 | 19 |  |  |  |  |
| 3.42 | 18 |  |  |  |  |
| 3.05 | 17 |  |  |  |  |
| 2.55 | 16 |  |  |  |  |
| 5.52 | 23 | 3.845 | 5.5 | 20.12 | 3.85 |
| 5.06 | 22 |  |  |  |  |
| 4.53 | 21 |  |  |  |  |
| 4.01 | 20 |  |  |  |  |
| 3.47 | 19 |  |  |  |  |
| 3.07 | 18 |  |  |  |  |
| 2.7 | 17 |  |  |  |  |
| 2.22 | 16 |  |  |  |  |
| 7.7 | 25 | 3.525 | 7.7 | 17.25 | 3.53 |
| 7.5 | 24 |  |  |  |  |
| 7.3 | 23 |  |  |  |  |
| 6.9 | 22 |  |  |  |  |
| 6.4 | 21 |  |  |  |  |
| 6.2 | 20 |  |  |  |  |
| 5.9 | 19 |  |  |  |  |
| 5.6 | 18 |  |  |  |  |
| 6.8 | 25 | 4.665 | 6.8 | 15.66 | 4.67 |
| 6.5 | 24 |  |  |  |  |
| 6.2 | 23 |  |  |  |  |
| 5.8 | 22 |  |  |  |  |
| 5.4 | 21 |  |  |  |  |
| 5 | 20 |  |  |  |  |
| 4.8 | 19 |  |  |  |  |
| 4.4 | 18 |  |  |  |  |
| 7.4 | 25 | 4.815 | 7.4 | 16.49 | 4.82 |
| 6.9 | 24 |  |  |  |  |
| 6.5 | 23 |  |  |  |  |
| 5.9 | 22 |  |  |  |  |
| 5.6 | 21 |  |  |  |  |
| 5.2 | 20 |  |  |  |  |
| 4.8 | 19 |  |  |  |  |
| 4.5 | 18 |  |  |  |  |
| 7.4 | 25 | 3.6 | 7.4 | 16.77 | 3.6 |
| 6.9 | 24 |  |  |  |  |
| 6.2 | 23 |  |  |  |  |
| 5.7 | 22 |  |  |  |  |
| 5.1 | 21 |  |  |  |  |
| 4.6 | 20 |  |  |  |  |
| 4 | 19 |  |  |  |  |
| 3.6 | 18 |  |  |  |  |
| 9.2 | 25 | 3.7 | 9.2 | 16.87 | 3.7 |
| 8.8 | 24 |  |  |  |  |
| 8.3 | 23 |  |  |  |  |
| 7.7 | 22 |  |  |  |  |
| 7.2 | 21 |  |  |  |  |
| 6.6 | 20 |  |  |  |  |
| 6.1 | 19 |  |  |  |  |
| 5.6 | 18 |  |  |  |  |
| 8.4 | 25 | 3.325 | 8.4 | 16.17 | 3.33 |
| 8.2 | 24 |  |  |  |  |
| 7.7 | 23 |  |  |  |  |
| 7.3 | 22 |  |  |  |  |
| 6.9 | 21 |  |  |  |  |
| 6.4 | 20 |  |  |  |  |
| 5.8 | 19 |  |  |  |  |
| 5.2 | 18 |  |  |  |  |
| 7.6 | 25 | 4.025 | 7.6 | 17.51 | 4.03 |
| 7.1 | 24 |  |  |  |  |
| 6.7 | 23 |  |  |  |  |
| 6.3 | 22 |  |  |  |  |
| 6 | 21 |  |  |  |  |
| 5.6 | 20 |  |  |  |  |
| 5.1 | 19 |  |  |  |  |
| 4.8 | 18 |  |  |  |  |
| 8 | 25 | 5.075 | 8 | 17.19 | 5.08 |
| 7.6 | 24 |  |  |  |  |
| 7.1 | 23 |  |  |  |  |
| 6.5 | 22 |  |  |  |  |
| 6 | 21 |  |  |  |  |
| 5.3 | 20 |  |  |  |  |
| 4.7 | 19 |  |  |  |  |
| 4.4 | 18 |  |  |  |  |
| 8.1 | 25 | 5.66 | 8.1 | 21.33 | 5.66 |
| 7.7 | 24 |  |  |  |  |
| 7.1 | 23 |  |  |  |  |
| 6.7 | 22 |  |  |  |  |
| 6.2 | 21 |  |  |  |  |
| 5.6 | 20 |  |  |  |  |
| 5.4 | 19 |  |  |  |  |
| 5.1 | 18 |  |  |  |  |
| 8 | 25 | 4.375 | 8 | 18.97 | 4.38 |
| 7.7 | 24 |  |  |  |  |
| 7.4 | 23 |  |  |  |  |
| 7.2 | 22 |  |  |  |  |
| 6.8 | 21 |  |  |  |  |
| 6.2 | 20 |  |  |  |  |
| 5.6 | 19 |  |  |  |  |
| 5.1 | 18 |  |  |  |  |
| 7.2 | 25 | 4.825 | 7.2 | 18.68 | 4.83 |
| 6.8 | 24 |  |  |  |  |
| 6.3 | 23 |  |  |  |  |
| 5.7 | 22 |  |  |  |  |
| 5.2 | 21 |  |  |  |  |
| 4.6 | 20 |  |  |  |  |
| 4 | 19 |  |  |  |  |
| 3.4 | 18 |  |  |  |  |
| 8.6 | 25 | 4.175 | 8.6 | 18.24 | 4.18 |
| 8 | 24 |  |  |  |  |
| 7.5 | 23 |  |  |  |  |
| 7.3 | 22 |  |  |  |  |
| 6.6 | 21 |  |  |  |  |
| 6.2 | 20 |  |  |  |  |
| 5.4 | 19 |  |  |  |  |
| 4.8 | 18 |  |  |  |  |
| 6.7 | 25 | 4.075 | 6.7 | 15.41 | 4.08 |
| 6.2 | 24 |  |  |  |  |
| 5.8 | 23 |  |  |  |  |
| 5.4 | 22 |  |  |  |  |
| 5.2 | 21 |  |  |  |  |
| 5 | 20 |  |  |  |  |
| 4.6 | 19 |  |  |  |  |
| 4.1 | 18 |  |  |  |  |
| 8.2 | 25 | 3.45 | 8.2 | 15.22 | 3.45 |
| 7.7 | 24 |  |  |  |  |
| 7.2 | 23 |  |  |  |  |
| 6.7 | 22 |  |  |  |  |
| 6.1 | 21 |  |  |  |  |
| 5.5 | 20 |  |  |  |  |
| 5 | 19 |  |  |  |  |
| 4.2 | 18 |  |  |  |  |
| 7.6 | 25 | 3.89 | 7.6 | 16.07 | 3.89 |
| 7.4 | 24 |  |  |  |  |
| 7.2 | 23 |  |  |  |  |
| 6.9 | 22 |  |  |  |  |
| 6.5 | 21 |  |  |  |  |
| 5.9 | 20 |  |  |  |  |
| 5.6 | 19 |  |  |  |  |
| 5.1 | 18 |  |  |  |  |
| 6.7 | 25 | 4.26 | 6.7 | 16.23 | 4.26 |
| 6.3 | 24 |  |  |  |  |
| 5.9 | 23 |  |  |  |  |
| 5.4 | 22 |  |  |  |  |
| 5 | 21 |  |  |  |  |
| 4.5 | 20 |  |  |  |  |
| 4.1 | 19 |  |  |  |  |
| 3.7 | 18 |  |  |  |  |
| 9.9 | 27 | 3.965 | 9.9 | 19.39 | 3.97 |
| 9.7 | 26 |  |  |  |  |
| 9.3 | 25 |  |  |  |  |
| 9 | 24 |  |  |  |  |
| 8.8 | 23 |  |  |  |  |
| 8.5 | 22 |  |  |  |  |
| 8.1 | 21 |  |  |  |  |
| 7.8 | 20 |  |  |  |  |
| 10.3 | 27 | 4.35 | 10.3 | 19.42 | 4.35 |
| 9.9 | 26 |  |  |  |  |
| 9.7 | 25 |  |  |  |  |
| 9.3 | 24 |  |  |  |  |
| 8.8 | 23 |  |  |  |  |
| 8.4 | 22 |  |  |  |  |
| 8.1 | 21 |  |  |  |  |
| 7.6 | 20 |  |  |  |  |
| 9.6 | 27 | 4.945 | 9.6 | 18.84 | 4.95 |
| 9.3 | 26 |  |  |  |  |
| 9 | 25 |  |  |  |  |
| 8.8 | 24 |  |  |  |  |
| 8.4 | 23 |  |  |  |  |
| 8 | 22 |  |  |  |  |
| 7.7 | 21 |  |  |  |  |
| 7.3 | 20 |  |  |  |  |
| 9.9 | 27 | 3.795 | 9.9 | 19.1 | 3.8 |
| 9.5 | 26 |  |  |  |  |
| 9.4 | 25 |  |  |  |  |
| 9.1 | 24 |  |  |  |  |
| 8.9 | 23 |  |  |  |  |
| 8.7 | 22 |  |  |  |  |
| 8.4 | 21 |  |  |  |  |
| 8.1 | 20 |  |  |  |  |
| 8.5 | 27 | 3.785 | 8.5 | 18.72 | 3.79 |
| 8.2 | 26 |  |  |  |  |
| 7.8 | 25 |  |  |  |  |
| 7.3 | 24 |  |  |  |  |
| 6.8 | 23 |  |  |  |  |
| 6.3 | 22 |  |  |  |  |
| 5.8 | 21 |  |  |  |  |
| 5.4 | 20 |  |  |  |  |
| 8.1 | 27 | 3.945 | 8.1 | 19.29 | 3.95 |
| 7.8 | 26 |  |  |  |  |
| 7.4 | 25 |  |  |  |  |
| 7 | 24 |  |  |  |  |
| 6.5 | 23 |  |  |  |  |
| 6.1 | 22 |  |  |  |  |
| 5.8 | 21 |  |  |  |  |
| 5.3 | 20 |  |  |  |  |
| 8.4 | 27 | 5.2 | 8.4 | 19.77 | 5.2 |
| 8.2 | 26 |  |  |  |  |
| 8 | 25 |  |  |  |  |
| 7.5 | 24 |  |  |  |  |
| 7.1 | 23 |  |  |  |  |
| 6.7 | 22 |  |  |  |  |
| 6.3 | 21 |  |  |  |  |
| 6 | 20 |  |  |  |  |
| 8.6 | 27 | 4.72 | 8.6 | 19.99 | 4.72 |
| 8.3 | 26 |  |  |  |  |
| 8.1 | 25 |  |  |  |  |
| 7.8 | 24 |  |  |  |  |
| 7.5 | 23 |  |  |  |  |
| 7.4 | 22 |  |  |  |  |
| 7.1 | 21 |  |  |  |  |
| 6.8 | 20 |  |  |  |  |
| 10.1 | 27 | 2.91 | 10.1 | 19.1 | 2.91 |
| 9.7 | 26 |  |  |  |  |
| 9.2 | 25 |  |  |  |  |
| 8.7 | 24 |  |  |  |  |
| 8 | 23 |  |  |  |  |
| 7.7 | 22 |  |  |  |  |
| 6.9 | 21 |  |  |  |  |
| 6.6 | 20 |  |  |  |  |
| 9.2 | 27 | 4.34 | 9.2 | 19.1 | 4.34 |
| 9.1 | 26 |  |  |  |  |
| 8.7 | 25 |  |  |  |  |
| 8.5 | 24 |  |  |  |  |
| 8 | 23 |  |  |  |  |
| 7.6 | 22 |  |  |  |  |
| 7 | 21 |  |  |  |  |
| 6.5 | 20 |  |  |  |  |
| 8.7 | 27 | 4 | 8.7 | 21.26 | 4 |
| 8.3 | 26 |  |  |  |  |
| 8.1 | 25 |  |  |  |  |
| 7.7 | 24 |  |  |  |  |
| 7.2 | 23 |  |  |  |  |
| 6.9 | 22 |  |  |  |  |
| 6.4 | 21 |  |  |  |  |
| 6 | 20 |  |  |  |  |
| 10.1 | 27 | 5.415 | 10.1 | 20.85 | 5.42 |
| 9.9 | 26 |  |  |  |  |
| 9.6 | 25 |  |  |  |  |
| 9 | 24 |  |  |  |  |
| 8.5 | 23 |  |  |  |  |
| 8.1 | 22 |  |  |  |  |
| 7.6 | 21 |  |  |  |  |
| 7.1 | 20 |  |  |  |  |
| 9 | 27 | 4.84 | 9 | 19.64 | 4.84 |
| 8.8 | 26 |  |  |  |  |
| 8.7 | 25 |  |  |  |  |
| 8.5 | 24 |  |  |  |  |
| 8.1 | 23 |  |  |  |  |
| 7.8 | 22 |  |  |  |  |
| 7.3 | 21 |  |  |  |  |
| 6.8 | 20 |  |  |  |  |
| 11.1 | 27 | 4.125 | 11.1 | 21.33 | 4.13 |
| 10.8 | 26 |  |  |  |  |
| 10.6 | 25 |  |  |  |  |
| 10 | 24 |  |  |  |  |
| 9.6 | 23 |  |  |  |  |
| 8.9 | 22 |  |  |  |  |
| 8.4 | 21 |  |  |  |  |
| 8.1 | 20 |  |  |  |  |
| 9.4 | 27 | 5.435 | 9.4 | 20.85 | 5.44 |
| 9.1 | 26 |  |  |  |  |
| 8.9 | 25 |  |  |  |  |
| 8.5 | 24 |  |  |  |  |
| 8.1 | 23 |  |  |  |  |
| 7.7 | 22 |  |  |  |  |
| 7.3 | 21 |  |  |  |  |
| 6.9 | 20 |  |  |  |  |
| 9 | 27 | 5.17 | 9 | 21.87 | 5.17 |
| 8.8 | 26 |  |  |  |  |
| 8.6 | 25 |  |  |  |  |
| 8.3 | 24 |  |  |  |  |
| 8.1 | 23 |  |  |  |  |
| 7.9 | 22 |  |  |  |  |
| 7.6 | 21 |  |  |  |  |
| 7.3 | 20 |  |  |  |  |
| 9.9 | 42 | 4.79 | 9.9 | 20.94 | 4.79 |
| 9.7 | 41 |  |  |  |  |
| 9.5 | 40 |  |  |  |  |
| 9.4 | 39 |  |  |  |  |
| 9.2 | 38 |  |  |  |  |
| 8.9 | 37 |  |  |  |  |
| 8.7 | 36 |  |  |  |  |
| 8.5 | 35 |  |  |  |  |
| 12 | 42 | 5.1 | 12 | 22.54 | 5.1 |
| 11.7 | 41 |  |  |  |  |
| 11.3 | 40 |  |  |  |  |
| 10.8 | 39 |  |  |  |  |
| 10.4 | 38 |  |  |  |  |
| 10 | 37 |  |  |  |  |
| 9.6 | 36 |  |  |  |  |
| 9.1 | 35 |  |  |  |  |
| 12.8 | 42 | 5.3 | 12.8 | 25.15 | 5.3 |
| 12.6 | 41 |  |  |  |  |
| 12.4 | 40 |  |  |  |  |
| 11.9 | 39 |  |  |  |  |
| 11.4 | 38 |  |  |  |  |
| 11.1 | 37 |  |  |  |  |
| 10.7 | 36 |  |  |  |  |
| 10.4 | 35 |  |  |  |  |
| 12.8 | 42 | 5.29 | 12.8 | 26.99 | 5.29 |
| 12.7 | 41 |  |  |  |  |
| 12.5 | 40 |  |  |  |  |
| 12.4 | 39 |  |  |  |  |
| 12.2 | 38 |  |  |  |  |
| 12 | 37 |  |  |  |  |
| 11.8 | 36 |  |  |  |  |
| 11.6 | 35 |  |  |  |  |
| 11.9 | 42 | 4.495 | 11.9 | 22.79 | 4.5 |
| 11.6 | 41 |  |  |  |  |
| 11.1 | 40 |  |  |  |  |
| 10.6 | 39 |  |  |  |  |
| 10.1 | 38 |  |  |  |  |
| 9.8 | 37 |  |  |  |  |
| 9.5 | 36 |  |  |  |  |
| 9.1 | 35 |  |  |  |  |
| 13.7 | 42 | 6.315 | 13.7 | 24.67 | 6.32 |
| 13.5 | 41 |  |  |  |  |
| 13 | 40 |  |  |  |  |
| 12.5 | 39 |  |  |  |  |
| 12.1 | 38 |  |  |  |  |
| 11.6 | 37 |  |  |  |  |
| 11.3 | 36 |  |  |  |  |
| 10.5 | 35 |  |  |  |  |
| 13.6 | 42 | 6.06 | 13.6 | 25.94 | 6.06 |
| 13.3 | 41 |  |  |  |  |
| 12.7 | 40 |  |  |  |  |
| 12.2 | 39 |  |  |  |  |
| 11.4 | 38 |  |  |  |  |
| 10.7 | 37 |  |  |  |  |
| 10.1 | 36 |  |  |  |  |
| 9.8 | 35 |  |  |  |  |
| 11.6 | 42 | 4.745 | 11.6 | 22.41 | 4.75 |
| 11.4 | 41 |  |  |  |  |
| 11 | 40 |  |  |  |  |
| 10.4 | 39 |  |  |  |  |
| 10 | 38 |  |  |  |  |
| 9.6 | 37 |  |  |  |  |
| 9.3 | 36 |  |  |  |  |
| 9.1 | 35 |  |  |  |  |
| 11.3 | 42 | 4.825 | 11.3 | 24.67 | 4.83 |
| 11.1 | 41 |  |  |  |  |
| 10.8 | 40 |  |  |  |  |
| 10.7 | 39 |  |  |  |  |
| 10.3 | 38 |  |  |  |  |
| 9.8 | 37 |  |  |  |  |
| 9.6 | 36 |  |  |  |  |
| 9.2 | 35 |  |  |  |  |
| 11.4 | 42 | 5.96 | 11.4 | 24.83 | 5.96 |
| 11 | 41 |  |  |  |  |
| 10.6 | 40 |  |  |  |  |
| 10.4 | 39 |  |  |  |  |
| 9.8 | 38 |  |  |  |  |
| 9.5 | 37 |  |  |  |  |
| 9 | 36 |  |  |  |  |
| 8.6 | 35 |  |  |  |  |
| 12.3 | 42 | 5.485 | 12.3 | 26.36 | 5.49 |
| 12.2 | 41 |  |  |  |  |
| 11.8 | 40 |  |  |  |  |
| 11.5 | 39 |  |  |  |  |
| 11.2 | 38 |  |  |  |  |
| 10.9 | 37 |  |  |  |  |
| 10.7 | 36 |  |  |  |  |
| 10.4 | 35 |  |  |  |  |
| 12.6 | 42 | 6.36 | 12.6 | 26.1 | 6.36 |
| 12.3 | 41 |  |  |  |  |
| 11.9 | 40 |  |  |  |  |
| 11.5 | 39 |  |  |  |  |
| 11 | 38 |  |  |  |  |
| 10.5 | 37 |  |  |  |  |
| 10.1 | 36 |  |  |  |  |
| 9.5 | 35 |  |  |  |  |
| 12 | 42 | 3.13 | 12 | 16.93 | 3.13 |
| 11.8 | 41 |  |  |  |  |
| 11.5 | 40 |  |  |  |  |
| 11.3 | 39 |  |  |  |  |
| 11.1 | 38 |  |  |  |  |
| 10.7 | 37 |  |  |  |  |
| 10.3 | 36 |  |  |  |  |
| 9.8 | 35 |  |  |  |  |
| 13 | 42 | 3 | 13 | 18.3 | 3 |
| 12.6 | 41 |  |  |  |  |
| 12.2 | 40 |  |  |  |  |
| 11.8 | 39 |  |  |  |  |
| 11.4 | 38 |  |  |  |  |
| 10.9 | 37 |  |  |  |  |
| 10.7 | 36 |  |  |  |  |
| 10.4 | 35 |  |  |  |  |
| 12.7 | 42 | 4.23 | 12.7 | 19.29 | 4.23 |
| 12.4 | 41 |  |  |  |  |
| 12.1 | 40 |  |  |  |  |
| 11.4 | 39 |  |  |  |  |
| 10.6 | 38 |  |  |  |  |
| 10.1 | 37 |  |  |  |  |
| 9.7 | 36 |  |  |  |  |
| 9.5 | 35 |  |  |  |  |
| 12.3 | 42 | 3.815 | 12.3 | 18.97 | 3.82 |
| 12.1 | 41 |  |  |  |  |
| 11.7 | 40 |  |  |  |  |
| 11.1 | 39 |  |  |  |  |
| 10.6 | 38 |  |  |  |  |
| 10.1 | 37 |  |  |  |  |
| 9.5 | 36 |  |  |  |  |
| 8.8 | 35 |  |  |  |  |
