# Supplementary material for: Impact of climate factors on height growth of Pinus sylvestris var. mongolica
Source: PLoS One. 2019 Mar 11;14(3):e0213509. doi: 10.1371/journal.pone.0213509 (PMC6411114; doi:10.1371/journal.pone.0213509)
Supplement: S3 Table — (DOCX) [file pone.0213509.s003.docx]

**Supporting Information to:**

**Impact of climate factors on height growth of *Pinus sylvestris* var. *mongolica***

Yanping Zhou, Zeyong Lei, Fengyan Zhou, Yangang Han, Deliang Yu, Yansong Zhang

**S3 Table. Values of validation data shown in Table 1.**

| h | t | H | D | CW |
| --- | --- | --- | --- | --- |
| 3.6 | 13 | 3.6 | 10.12 | 2.78 |
| 3.3 | 12 |  |  |  |
| 2.8 | 11 |  |  |  |
| 2.3 | 10 |  |  |  |
| 2.1 | 9 |  |  |  |
| 1.6 | 8 |  |  |  |
| 1.1 | 7 |  |  |  |
| 0.9 | 6 |  |  |  |
| 3.2 | 13 | 3.2 | 10.38 | 2.53 |
| 2.9 | 12 |  |  |  |
| 2.4 | 11 |  |  |  |
| 2.1 | 10 |  |  |  |
| 1.6 | 9 |  |  |  |
| 1.3 | 8 |  |  |  |
| 1 | 7 |  |  |  |
| 0.4 | 6 |  |  |  |
| 2.7 | 13 | 2.7 | 15.85 | 2 |
| 2.2 | 12 |  |  |  |
| 1.7 | 11 |  |  |  |
| 1.3 | 10 |  |  |  |
| 0.9 | 9 |  |  |  |
| 0.7 | 8 |  |  |  |
| 0.4 | 7 |  |  |  |
| 0.3 | 6 |  |  |  |
| 3.4 | 13 | 3.4 | 9.49 | 2.58 |
| 2.9 | 12 |  |  |  |
| 2.4 | 11 |  |  |  |
| 1.9 | 10 |  |  |  |
| 1.4 | 9 |  |  |  |
| 1 | 8 |  |  |  |
| 0.7 | 7 |  |  |  |
| 0.5 | 6 |  |  |  |
| 5.18 | 23 | 5.2 | 19.1 | 4.47 |
| 4.85 | 22 |  |  |  |
| 4.42 | 21 |  |  |  |
| 3.88 | 20 |  |  |  |
| 3.34 | 19 |  |  |  |
| 3 | 18 |  |  |  |
| 2.76 | 17 |  |  |  |
| 2.35 | 16 |  |  |  |
| 5.15 | 23 | 5.2 | 16.3 | 3.95 |
| 4.52 | 22 |  |  |  |
| 4.03 | 21 |  |  |  |
| 3.52 | 20 |  |  |  |
| 3.16 | 19 |  |  |  |
| 2.83 | 18 |  |  |  |
| 2.43 | 17 |  |  |  |
| 2.04 | 16 |  |  |  |
| 5.96 | 23 | 6 | 17.7 | 3.86 |
| 5.66 | 22 |  |  |  |
| 5.03 | 21 |  |  |  |
| 4.4 | 20 |  |  |  |
| 3.81 | 19 |  |  |  |
| 3.3 | 18 |  |  |  |
| 2.99 | 17 |  |  |  |
| 2.5 | 16 |  |  |  |
| 5.39 | 23 | 5.4 | 17.44 | 4.69 |
| 5.07 | 22 |  |  |  |
| 4.48 | 21 |  |  |  |
| 4.08 | 20 |  |  |  |
| 3.61 | 19 |  |  |  |
| 3.2 | 18 |  |  |  |
| 2.7 | 17 |  |  |  |
| 2.35 | 16 |  |  |  |
| 9.1 | 27 | 9.1 | 18.65 | 3.75 |
| 8.8 | 26 |  |  |  |
| 8.5 | 25 |  |  |  |
| 8.1 | 24 |  |  |  |
| 7.9 | 23 |  |  |  |
| 7.6 | 22 |  |  |  |
| 7 | 21 |  |  |  |
| 6.5 | 20 |  |  |  |
| 7.8 | 27 | 7.8 | 18.14 | 3.2 |
| 7.7 | 26 |  |  |  |
| 7.4 | 25 |  |  |  |
| 7.2 | 24 |  |  |  |
| 7 | 23 |  |  |  |
| 6.8 | 22 |  |  |  |
| 6.6 | 21 |  |  |  |
| 6.4 | 20 |  |  |  |
| 9.2 | 27 | 9.2 | 17.48 | 4.73 |
| 8.9 | 26 |  |  |  |
| 8.6 | 25 |  |  |  |
| 8.4 | 24 |  |  |  |
| 8.2 | 23 |  |  |  |
| 7.9 | 22 |  |  |  |
| 7.7 | 21 |  |  |  |
| 7.4 | 20 |  |  |  |
| 9.1 | 27 | 9.1 | 17.63 | 3.5 |
| 8.8 | 26 |  |  |  |
| 8.3 | 25 |  |  |  |
| 7.8 | 24 |  |  |  |
| 7.5 | 23 |  |  |  |
| 7.3 | 22 |  |  |  |
| 7.2 | 21 |  |  |  |
| 6.8 | 20 |  |  |  |
| 11.1 | 42 | 11.1 | 20.37 | 4.29 |
| 10.8 | 41 |  |  |  |
| 10.5 | 40 |  |  |  |
| 10 | 39 |  |  |  |
| 9.7 | 38 |  |  |  |
| 9.3 | 37 |  |  |  |
| 8.7 | 36 |  |  |  |
| 8.4 | 35 |  |  |  |
| 11.9 | 42 | 11.9 | 20.47 | 4.34 |
| 11.7 | 41 |  |  |  |
| 11.3 | 40 |  |  |  |
| 10.7 | 39 |  |  |  |
| 10.3 | 38 |  |  |  |
| 10 | 37 |  |  |  |
| 9.6 | 36 |  |  |  |
| 9.2 | 35 |  |  |  |
| 12.3 | 42 | 12.3 | 19.48 | 4.69 |
| 11.9 | 41 |  |  |  |
| 11.5 | 40 |  |  |  |
| 10.9 | 39 |  |  |  |
| 10.4 | 38 |  |  |  |
| 9.8 | 37 |  |  |  |
| 9.4 | 36 |  |  |  |
| 9.1 | 35 |  |  |  |
| 11.5 | 42 | 11.5 | 21.23 | 3.89 |
| 11.2 | 41 |  |  |  |
| 10.7 | 40 |  |  |  |
| 10.3 | 39 |  |  |  |
| 9.9 | 38 |  |  |  |
| 9.7 | 37 |  |  |  |
| 9.3 | 36 |  |  |  |
| 8.8 | 35 |  |  |  |
