# Supplementary material for: Impact of climate factors on height growth of Pinus sylvestris var. mongolica
Source: PLoS One. 2019 Mar 11;14(3):e0213509. doi: 10.1371/journal.pone.0213509 (PMC6411114; doi:10.1371/journal.pone.0213509)
Supplement: S4 Table — (DOCX) [file pone.0213509.s004.docx]

**Supporting Information to:**

**Impact of climate factors on height growth of *Pinus sylvestris* var. *mongolica***

Yanping Zhou, Zeyong Lei, Fengyan Zhou, Yangang Han, Deliang Yu, Yansong Zhang

**S4 Table. Origin meteorological observations shown in Table 2 and Table 3.**

|  | MAT | MAP | MTCM | MTWM | MTM | PM | GST | GSP | GDD | AIK | PGP | PNP | PL |
| --- | --- | --- | --- | --- | --- | --- | --- | --- | --- | --- | --- | --- | --- |
| 1974 | 7.1 | 480.2 | -11.76 | 25 | 15.73 | 39.8 | 21.31 | 382.3 | 2313.5 | 4.4 | 434.7 | 70 | 513.3 |
| 1975 | 8.22 | 635.4 | -9.88 | 23.94 | 16.95 | 50.6 | 20.76 | 582.4 | 2413.4 | 5.87 | 382.3 | 102.1 | 480.2 |
| 1976 | 6.49 | 502.4 | -9.94 | 22.38 | 15.55 | 87.8 | 18.88 | 356.1 | 2126.8 | 5.32 | 582.4 | 84.3 | 635.4 |
| 1977 | 7 | 659.2 | -15.79 | 24.75 | 16.45 | 27.2 | 20.04 | 538.8 | 2303.7 | 6.34 | 356.1 | 104.3 | 502.4 |
| 1978 | 7.61 | 506.4 | -8.53 | 23.68 | 15.16 | 32 | 19.78 | 450.6 | 2327 | 5.05 | 538.8 | 127.3 | 659.2 |
| 1979 | 7.7 | 695 | -10.89 | 23.62 | 16.72 | 47.3 | 19.79 | 592.2 | 2265.6 | 6.97 | 450.6 | 83.5 | 506.4 |
| 1980 | 6.66 | 392.5 | -13.23 | 23.4 | 16.02 | 14.8 | 19.92 | 338.5 | 2284.2 | 4.01 | 592.2 | 68.9 | 695 |
| 1981 | 7.39 | 436.4 | -13.34 | 25.08 | 16.23 | 22.4 | 20.56 | 352.6 | 2383 | 4.06 | 338.5 | 95.1 | 392.5 |
| 1982 | 8.03 | 331.4 | -12.23 | 24.05 | 16.2 | 44.5 | 20.73 | 262.1 | 2408.8 | 3.04 | 352.6 | 53.5 | 436.4 |
| 1983 | 6.84 | 428.3 | -11.1 | 22.65 | 16.44 | 33.5 | 20.04 | 295.7 | 2180.13 | 4.25 | 262.1 | 149.6 | 331.4 |
| 1984 | 5.7 | 627.1 | -15.61 | 23.43 | 17.2 | 27.8 | 19.72 | 579.6 | 2160.2 | 6.43 | 295.7 | 44 | 428.3 |
| 1985 | 5.3 | 417 | -17.02 | 22.93 | 16.02 | 15.8 | 19.2 | 355.5 | 2075.13 | 4.25 | 579.6 | 65.1 | 627.1 |
| 1986 | 5.8 | 572.1 | -14.73 | 21.97 | 16.14 | 0 | 19.16 | 515.6 | 2026.2 | 6.07 | 355.5 | 54 | 417 |
| 1987 | 5.84 | 419.5 | -15.5 | 22.52 | 14.96 | 77.15 | 19.17 | 398.05 | 2006.4 | 4.29 | 515.6 | 37.2 | 572.1 |
| 1988 | 6.63 | 436.45 | -12.54 | 24.21 | 14.77 | 61.65 | 20.24 | 401.15 | 2110.38 | 4.21 | 398.05 | 18.15 | 419.5 |
| 1989 | 6.6 | 556.9 | -14.39 | 22.04 | 16.46 | 76.1 | 19.21 | 475.7 | 2097.38 | 5.61 | 401.15 | 80.6 | 436.45 |
| 1990 | 7.13 | 573.2 | -15.69 | 22.93 | 14.99 | 62.8 | 19.83 | 460.2 | 2122.675 | 5.62 | 475.7 | 118.8 | 556.9 |
| 1991 | 6.25 | 568.9 | -13.95 | 21.95 | 15.3 | 27.8 | 19.62 | 502.2 | 2110.05 | 5.73 | 460.2 | 31.2 | 573.2 |
| 1992 | 6.48 | 660.4 | -10.3 | 23.65 | 15.72 | 33.8 | 19.03 | 517.6 | 2060.875 | 6.91 | 502.2 | 68.6 | 568.9 |
| 1993 | 6.32 | 536.4 | -13.12 | 22.22 | 15.92 | 16.2 | 19.16 | 456.9 | 2054.575 | 5.68 | 517.6 | 162.4 | 660.4 |
| 1994 | 6.83 | 662 | -14.15 | 25.12 | 14.31 | 70.3 | 20.27 | 641.9 | 2086.725 | 6.34 | 456.9 | 51.8 | 536.4 |
| 1995 | 6.71 | 503.6 | -12.3 | 21.91 | 14.48 | 24.9 | 19.14 | 445 | 1982.35 | 5.15 | 641.9 | 28.2 | 662 |
| 1996 | 6.14 | 288.1 | -14.31 | 23.3 | 17.05 | 42.2 | 19.83 | 248.1 | 2151.8 | 2.9 | 445 | 65.1 | 503.6 |
| 1997 | 6.95 | 435.3 | -15.36 | 25 | 15.33 | 40.8 | 20.17 | 409.6 | 2124.325 | 4.3 | 248.1 | 31.9 | 288.1 |
| 1998 | 7.63 | 423.9 | -14.24 | 23.17 | 16.73 | 16.5 | 19.87 | 377.1 | 2194.625 | 3.95 | 409.6 | 31 | 435.3 |
| 1999 | 6.58 | 369.3 | -10.96 | 24.99 | 16.16 | 22 | 20.13 | 295 | 2194.45 | 3.72 | 377.1 | 43.7 | 423.9 |
| 2000 | 6.44 | 377.1 | -17.49 | 26.13 | 17.37 | 44.5 | 21.9 | 276.8 | 2403.45 | 3.43 | 295 | 108 | 369.3 |
| 2001 | 6.45 | 311.4 | -19.11 | 24.53 | 17.1 | 27.4 | 20.74 | 294.1 | 2253.025 | 2.88 | 276.8 | 57.2 | 377.1 |
| 2002 | 7.33 | 289.6 | -10.46 | 24.42 | 18.83 | 9.5 | 20.6 | 228 | 2370.695 | 2.82 | 294.1 | 53.8 | 311.4 |
| 2003 | 7.47 | 432.6 | -12.91 | 22.79 | 17.24 | 3.6 | 19.97 | 377.3 | 2200.53 | 4.15 | 228 | 22.4 | 289.6 |
| 2004 | 7.56 | 474.4 | -12.1 | 22.47 | 16.21 | 40.5 | 19.83 | 388.2 | 2108.57 | 4.52 | 377.3 | 59.1 | 432.6 |
| 2005 | 6.6 | 567.6 | -12.37 | 22.98 | 14.67 | 45.6 | 19.52 | 515.3 | 2064.9 | 5.4 | 388.2 | 106.7 | 474.4 |
| 2006 | 6.58 | 346.6 | -13.14 | 22.88 | 16.52 | 40.5 | 19.85 | 277.4 | 2184.2 | 3.46 | 515.3 | 66.5 | 567.6 |
| 2007 | 7.87 | 345.2 | -9.68 | 23.15 | 17.17 | 35.4 | 20.83 | 274 | 2246.53 | 3.26 | 277.4 | 64.2 | 346.6 |
| 2008 | 7.17 | 516.8 | -14.38 | 23.36 | 15.14 | 60.8 | 19.48 | 459.1 | 2080.23 | 5.02 | 274 | 71.4 | 345.2 |
| 2009 | 6.47 | 285.9 | -12.26 | 22.67 | 17.84 | 17.3 | 19.8 | 166.6 | 2219.68 | 2.78 | 459.1 | 101.4 | 516.8 |
| 2010 | 5.99 | 689.2 | -13.64 | 23.61 | 15.61 | 105.3 | 19.95 | 525.6 | 2100.33 | 7.12 | 166.6 | 99.8 | 285.9 |
| 2011 | 7.9 | 485 | -13.29 | 23.93 | 17.12 | 54.5 | 20.39 | 431.7 | 2357.6 | 4.51 | 525.6 | 113.7 | 689.2 |
| 2012 | 7.34 | 713.2 | -13.54 | 23.84 | 18.25 | 33 | 20.85 | 517 | 2426.9 | 6.58 | 431.7 | 178.4 | 485 |
| 2013 | 7.78 | 595.2 | -12.91 | 24.46 | 18.51 | 12.7 | 21.24 | 459.7 | 2487.7 | 5.6 | 517 | 116.4 | 713.2 |
| 2014 | 8.99 | 398.6 | -8.02 | 24.08 | 17.14 | 57.7 | 20.61 | 360.8 | 2390.9 | 3.61 | 459.7 | 93.9 | 595.2 |
| 2015 | 8.71 | 337.1 | -9.2 | 23.81 | 17.49 | 60.7 | 20.99 | 263.4 | 2471.3 | 3.04 | 360.8 | 57.7 | 398.6 |
