# Supplementary material for: Impact of climate factors on height growth of Pinus sylvestris var. mongolica
Source: PLoS One. 2019 Mar 11;14(3):e0213509. doi: 10.1371/journal.pone.0213509 (PMC6411114; doi:10.1371/journal.pone.0213509)
Supplement: S5 Table — (DOCX) [file pone.0213509.s005.docx]

**Supporting Information to:**

**Impact of climate factors on height growth of *Pinus sylvestris* var. *mongolica***

Yanping Zhou, Zeyong Lei, Fengyan Zhou, Yangang Han, Deliang Yu, Yansong Zhang

**S5 Table. Values for calculating testing indices shown in Table 4.**

| year | group | h | t | MTCM | MTM | PNP | PGP |
| --- | --- | --- | --- | --- | --- | --- | --- |
| 2015 | 1 | 4.4 | 13 | -12.11 | 16.84 | 88.60 | 383.10 |
| 2014 | 1 | 4 | 12 | -12.35 | 16.79 | 91.20 | 385.00 |
| 2013 | 1 | 3.6 | 11 | -12.75 | 16.75 | 90.90 | 378.20 |
| 2012 | 1 | 3.2 | 10 | -12.73 | 16.58 | 88.40 | 364.30 |
| 2011 | 1 | 2.5 | 9 | -12.64 | 16.39 | 78.40 | 356.80 |
| 2010 | 1 | 2 | 8 | -12.56 | 16.30 | 73.90 | 335.70 |
| 2009 | 1 | 1.6 | 7 | -12.41 | 16.40 | 70.20 | 359.90 |
| 2008 | 1 | 1.2 | 6 | -12.43 | 16.16 | 65.10 | 343.40 |
| 2015 | 2 | 4.4 | 13 | -12.11 | 16.84 | 88.60 | 383.10 |
| 2014 | 2 | 3.9 | 12 | -12.35 | 16.79 | 91.20 | 385.00 |
| 2013 | 2 | 3.4 | 11 | -12.75 | 16.75 | 90.90 | 378.20 |
| 2012 | 2 | 2.8 | 10 | -12.73 | 16.58 | 88.40 | 364.30 |
| 2011 | 2 | 2.1 | 9 | -12.64 | 16.39 | 78.40 | 356.80 |
| 2010 | 2 | 1.7 | 8 | -12.56 | 16.30 | 73.90 | 335.70 |
| 2009 | 2 | 1.4 | 7 | -12.41 | 16.40 | 70.20 | 359.90 |
| 2008 | 2 | 1 | 6 | -12.43 | 16.16 | 65.10 | 343.40 |
| 2015 | 3 | 4.1 | 13 | -12.11 | 16.84 | 88.60 | 383.10 |
| 2014 | 3 | 3.6 | 12 | -12.35 | 16.79 | 91.20 | 385.00 |
| 2013 | 3 | 3 | 11 | -12.75 | 16.75 | 90.90 | 378.20 |
| 2012 | 3 | 2.5 | 10 | -12.73 | 16.58 | 88.40 | 364.30 |
| 2011 | 3 | 1.9 | 9 | -12.64 | 16.39 | 78.40 | 356.80 |
| 2010 | 3 | 1.5 | 8 | -12.56 | 16.30 | 73.90 | 335.70 |
| 2009 | 3 | 1.2 | 7 | -12.41 | 16.40 | 70.20 | 359.90 |
| 2008 | 3 | 0.9 | 6 | -12.43 | 16.16 | 65.10 | 343.40 |
| 2015 | 4 | 4.4 | 13 | -12.11 | 16.84 | 88.60 | 383.10 |
| 2014 | 4 | 3.8 | 12 | -12.35 | 16.79 | 91.20 | 385.00 |
| 2013 | 4 | 3.2 | 11 | -12.75 | 16.75 | 90.90 | 378.20 |
| 2012 | 4 | 2.6 | 10 | -12.73 | 16.58 | 88.40 | 364.30 |
| 2011 | 4 | 2.1 | 9 | -12.64 | 16.39 | 78.40 | 356.80 |
| 2010 | 4 | 1.6 | 8 | -12.56 | 16.30 | 73.90 | 335.70 |
| 2009 | 4 | 1.3 | 7 | -12.41 | 16.40 | 70.20 | 359.90 |
| 2008 | 4 | 0.9 | 6 | -12.43 | 16.16 | 65.10 | 343.40 |
| 2015 | 5 | 5.8 | 23 | -13.00 | 16.62 | 77.60 | 388.80 |
| 2014 | 5 | 5.3 | 22 | -13.17 | 16.58 | 78.50 | 390.10 |
| 2013 | 5 | 4.8 | 21 | -13.42 | 16.55 | 77.80 | 386.80 |
| 2012 | 5 | 4.4 | 20 | -13.44 | 16.45 | 75.80 | 380.30 |
| 2011 | 5 | 3.9 | 19 | -13.44 | 16.36 | 70.40 | 377.60 |
| 2010 | 5 | 3.4 | 18 | -13.44 | 16.32 | 68.00 | 369.30 |
| 2009 | 5 | 3.1 | 17 | -13.43 | 16.36 | 66.20 | 381.30 |
| 2008 | 5 | 2.6 | 16 | -13.51 | 16.26 | 64.00 | 376.40 |
| 2015 | 6 | 7.3 | 25 | -12.93 | 16.53 | 75.40 | 396.20 |
| 2014 | 6 | 7 | 24 | -13.08 | 16.49 | 76.10 | 397.70 |
| 2013 | 6 | 6.6 | 23 | -13.30 | 16.46 | 75.30 | 395.00 |
| 2012 | 6 | 6.1 | 22 | -13.32 | 16.37 | 73.50 | 389.40 |
| 2011 | 6 | 5.6 | 21 | -13.31 | 16.28 | 68.50 | 387.40 |
| 2010 | 6 | 5.3 | 20 | -13.31 | 16.24 | 66.20 | 380.50 |
| 2009 | 6 | 4.9 | 19 | -13.29 | 16.27 | 64.50 | 391.80 |
| 2008 | 6 | 4.5 | 18 | -13.35 | 16.18 | 62.40 | 388.00 |
| 2015 | 7 | 8.3 | 25 | -12.93 | 16.53 | 75.40 | 396.20 |
| 2014 | 7 | 7.9 | 24 | -13.08 | 16.49 | 76.10 | 397.70 |
| 2013 | 7 | 7.5 | 23 | -13.30 | 16.46 | 75.30 | 395.00 |
| 2012 | 7 | 7 | 22 | -13.32 | 16.37 | 73.50 | 389.40 |
| 2011 | 7 | 6.5 | 21 | -13.31 | 16.28 | 68.50 | 387.40 |
| 2010 | 7 | 6 | 20 | -13.31 | 16.24 | 66.20 | 380.50 |
| 2009 | 7 | 5.4 | 19 | -13.29 | 16.27 | 64.50 | 391.80 |
| 2008 | 7 | 5 | 18 | -13.35 | 16.18 | 62.40 | 388.00 |
| 2015 | 8 | 8 | 25 | -12.93 | 16.53 | 75.40 | 396.20 |
| 2014 | 8 | 7.6 | 24 | -13.08 | 16.49 | 76.10 | 397.70 |
| 2013 | 8 | 7.1 | 23 | -13.30 | 16.46 | 75.30 | 395.00 |
| 2012 | 8 | 6.7 | 22 | -13.32 | 16.37 | 73.50 | 389.40 |
| 2011 | 8 | 6.2 | 21 | -13.31 | 16.28 | 68.50 | 387.40 |
| 2010 | 8 | 5.7 | 20 | -13.31 | 16.24 | 66.20 | 380.50 |
| 2009 | 8 | 5.1 | 19 | -13.29 | 16.27 | 64.50 | 391.80 |
| 2008 | 8 | 4.6 | 18 | -13.35 | 16.18 | 62.40 | 388.00 |
| 2015 | 9 | 7.3 | 25 | -12.93 | 16.53 | 75.40 | 396.20 |
| 2014 | 9 | 6.9 | 24 | -13.08 | 16.49 | 76.10 | 397.70 |
| 2013 | 9 | 6.5 | 23 | -13.30 | 16.46 | 75.30 | 395.00 |
| 2012 | 9 | 6.1 | 22 | -13.32 | 16.37 | 73.50 | 389.40 |
| 2011 | 9 | 5.7 | 21 | -13.31 | 16.28 | 68.50 | 387.40 |
| 2010 | 9 | 5.2 | 20 | -13.31 | 16.24 | 66.20 | 380.50 |
| 2009 | 9 | 4.8 | 19 | -13.29 | 16.27 | 64.50 | 391.80 |
| 2008 | 9 | 4.3 | 18 | -13.35 | 16.18 | 62.40 | 388.00 |
| 2015 | 10 | 9.9 | 27 | -13.08 | 16.47 | 77.20 | 399.30 |
| 2014 | 10 | 9.6 | 26 | -13.23 | 16.43 | 77.90 | 400.80 |
| 2013 | 10 | 9.4 | 25 | -13.44 | 16.40 | 77.30 | 398.50 |
| 2012 | 10 | 9.1 | 24 | -13.46 | 16.31 | 75.70 | 393.50 |
| 2011 | 10 | 8.7 | 23 | -13.46 | 16.23 | 71.20 | 391.90 |
| 2010 | 10 | 8.4 | 22 | -13.47 | 16.19 | 69.30 | 385.80 |
| 2009 | 10 | 8.1 | 21 | -13.46 | 16.22 | 67.80 | 396.20 |
| 2008 | 10 | 7.7 | 20 | -13.52 | 16.14 | 66.10 | 393.10 |
| 2015 | 11 | 8.4 | 27 | -13.08 | 16.47 | 77.20 | 399.30 |
| 2014 | 11 | 8.1 | 26 | -13.23 | 16.43 | 77.90 | 400.80 |
| 2013 | 11 | 7.8 | 25 | -13.44 | 16.40 | 77.30 | 398.50 |
| 2012 | 11 | 7.4 | 24 | -13.46 | 16.31 | 75.70 | 393.50 |
| 2011 | 11 | 7 | 23 | -13.46 | 16.23 | 71.20 | 391.90 |
| 2010 | 11 | 6.6 | 22 | -13.47 | 16.19 | 69.30 | 385.80 |
| 2009 | 11 | 6.3 | 21 | -13.46 | 16.22 | 67.80 | 396.20 |
| 2008 | 11 | 5.9 | 20 | -13.52 | 16.14 | 66.10 | 393.10 |
| 2015 | 12 | 9.5 | 27 | -13.08 | 16.47 | 77.20 | 399.30 |
| 2014 | 12 | 9.3 | 26 | -13.23 | 16.43 | 77.90 | 400.80 |
| 2013 | 12 | 8.9 | 25 | -13.44 | 16.40 | 77.30 | 398.50 |
| 2012 | 12 | 8.5 | 24 | -13.46 | 16.31 | 75.70 | 393.50 |
| 2011 | 12 | 7.9 | 23 | -13.46 | 16.23 | 71.20 | 391.90 |
| 2010 | 12 | 7.6 | 22 | -13.47 | 16.19 | 69.30 | 385.80 |
| 2009 | 12 | 7 | 21 | -13.46 | 16.22 | 67.80 | 396.20 |
| 2008 | 12 | 6.6 | 20 | -13.52 | 16.14 | 66.10 | 393.10 |
| 2015 | 13 | 9.6 | 27 | -13.08 | 16.47 | 77.20 | 399.30 |
| 2014 | 13 | 9.4 | 26 | -13.23 | 16.43 | 77.90 | 400.80 |
| 2013 | 13 | 9.2 | 25 | -13.44 | 16.40 | 77.30 | 398.50 |
| 2012 | 13 | 8.8 | 24 | -13.46 | 16.31 | 75.70 | 393.50 |
| 2011 | 13 | 8.5 | 23 | -13.46 | 16.23 | 71.20 | 391.90 |
| 2010 | 13 | 8.1 | 22 | -13.47 | 16.19 | 69.30 | 385.80 |
| 2009 | 13 | 7.7 | 21 | -13.46 | 16.22 | 67.80 | 396.20 |
| 2008 | 13 | 7.3 | 20 | -13.52 | 16.14 | 66.10 | 393.10 |
| 2015 | 14 | 11.9 | 42 | -12.98 | 16.31 | 77.20 | 409.9 |
| 2014 | 14 | 11.7 | 41 | -13.08 | 16.29 | 77.60 | 411.1 |
| 2013 | 14 | 11.4 | 40 | -13.2 | 16.26 | 77.20 | 409.9 |
| 2012 | 14 | 11.1 | 39 | -13.21 | 16.21 | 76.20 | 407.2 |
| 2011 | 14 | 10.8 | 38 | -13.2 | 16.15 | 73.50 | 406.5 |
| 2010 | 14 | 10.5 | 37 | -13.2 | 16.13 | 72.50 | 403.3 |
| 2009 | 14 | 10.2 | 36 | -13.19 | 16.14 | 71.70 | 409.9 |
| 2008 | 14 | 9.9 | 35 | -13.21 | 16.09 | 70.80 | 408.5 |
| 2015 | 15 | 12.7 | 42 | -12.98 | 16.31 | 77.20 | 409.9 |
| 2014 | 15 | 12.5 | 41 | -13.08 | 16.29 | 77.60 | 411.1 |
| 2013 | 15 | 12 | 40 | -13.2 | 16.26 | 77.20 | 409.9 |
| 2012 | 15 | 11.4 | 39 | -13.21 | 16.21 | 76.20 | 407.2 |
| 2011 | 15 | 10.9 | 38 | -13.2 | 16.15 | 73.50 | 406.5 |
| 2010 | 15 | 10.4 | 37 | -13.2 | 16.13 | 72.50 | 403.3 |
| 2009 | 15 | 10.1 | 36 | -13.19 | 16.14 | 71.70 | 409.9 |
| 2008 | 15 | 9.6 | 35 | -13.21 | 16.09 | 70.80 | 408.5 |
| 2015 | 16 | 11.9 | 42 | -12.98 | 16.31 | 77.20 | 409.9 |
| 2014 | 16 | 11.7 | 41 | -13.08 | 16.29 | 77.60 | 411.1 |
| 2013 | 16 | 11.3 | 40 | -13.2 | 16.26 | 77.20 | 409.9 |
| 2012 | 16 | 11 | 39 | -13.21 | 16.21 | 76.20 | 407.2 |
| 2011 | 16 | 10.6 | 38 | -13.2 | 16.15 | 73.50 | 406.5 |
| 2010 | 16 | 10.2 | 37 | -13.2 | 16.13 | 72.50 | 403.3 |
| 2009 | 16 | 9.9 | 36 | -13.19 | 16.14 | 71.70 | 409.9 |
| 2008 | 16 | 9.4 | 35 | -13.21 | 16.09 | 70.80 | 408.5 |
| 2015 | 17 | 12.5 | 42 | -12.98 | 16.31 | 77.20 | 409.9 |
| 2014 | 17 | 12.2 | 41 | -13.08 | 16.29 | 77.60 | 411.1 |
| 2013 | 17 | 11.9 | 40 | -13.2 | 16.26 | 77.20 | 409.9 |
| 2012 | 17 | 11.4 | 39 | -13.21 | 16.21 | 76.20 | 407.2 |
| 2011 | 17 | 10.9 | 38 | -13.2 | 16.15 | 73.50 | 406.5 |
| 2010 | 17 | 10.5 | 37 | -13.2 | 16.13 | 72.50 | 403.3 |
| 2009 | 17 | 10.1 | 36 | -13.19 | 16.14 | 71.70 | 409.9 |
| 2008 | 17 | 9.6 | 35 | -13.21 | 16.09 | 70.80 | 408.5 |
