# Supplementary material for: Impact of climate factors on height growth of Pinus sylvestris var. mongolica
Source: PLoS One. 2019 Mar 11;14(3):e0213509. doi: 10.1371/journal.pone.0213509 (PMC6411114; doi:10.1371/journal.pone.0213509)
Supplement: S6 Table — (DOCX) [file pone.0213509.s006.docx]

**Supporting Information to:**

**Impact of climate factors on height growth of *Pinus sylvestris* var. *mongolica***

Yanping Zhou, Zeyong Lei, Fengyan Zhou, Yangang Han, Deliang Yu, Yansong Zhang

**S6 Table. Values for model fitting and parameters estimate shown in Table 4.**

| t | year | h | MTCM | MTM | PNP | PGP |
| --- | --- | --- | --- | --- | --- | --- |
| 13 | 2015 | 3.2 | -12.11 | 16.84 | 88.60 | 383.1 |
| 12 | 2014 | 2.8 | -12.35 | 16.79 | 91.20 | 385 |
| 11 | 2013 | 2.3 | -12.75 | 16.75 | 90.90 | 378.2 |
| 10 | 2012 | 1.9 | -12.73 | 16.58 | 88.40 | 364.3 |
| 9 | 2011 | 1.5 | -12.64 | 16.39 | 78.40 | 356.8 |
| 8 | 2010 | 1.2 | -12.56 | 16.3 | 73.90 | 335.7 |
| 7 | 2009 | 0.8 | -12.41 | 16.4 | 70.20 | 359.9 |
| 6 | 2008 | 0.5 | -12.43 | 16.16 | 65.10 | 343.4 |
| 23 | 2015 | 5.4 | -13 | 16.62 | 77.60 | 388.8 |
| 22 | 2014 | 5 | -13.17 | 16.58 | 78.50 | 390.1 |
| 21 | 2013 | 4.5 | -13.42 | 16.55 | 77.80 | 386.8 |
| 20 | 2012 | 4 | -13.44 | 16.45 | 75.80 | 380.3 |
| 19 | 2011 | 3.5 | -13.44 | 16.36 | 70.40 | 377.6 |
| 18 | 2010 | 3.1 | -13.44 | 16.32 | 68.00 | 369.3 |
| 17 | 2009 | 2.7 | -13.43 | 16.36 | 66.20 | 381.3 |
| 16 | 2008 | 2.3 | -13.51 | 16.26 | 64.00 | 376.4 |
| 27 | 2015 | 8.8 | -13.08 | 16.47 | 77.20 | 399.3 |
| 26 | 2014 | 8.6 | -13.23 | 16.43 | 77.90 | 400.8 |
| 25 | 2013 | 8.2 | -13.44 | 16.4 | 77.30 | 398.5 |
| 24 | 2012 | 7.9 | -13.46 | 16.31 | 75.70 | 393.5 |
| 23 | 2011 | 7.7 | -13.46 | 16.23 | 71.20 | 391.9 |
| 22 | 2010 | 7.4 | -13.47 | 16.19 | 69.30 | 385.8 |
| 21 | 2009 | 7.1 | -13.46 | 16.22 | 67.80 | 396.2 |
| 20 | 2008 | 6.8 | -13.52 | 16.14 | 66.10 | 393.1 |
| 42 | 2015 | 11.7 | -12.98 | 16.31 | 77.20 | 409.9 |
| 41 | 2014 | 11.4 | -13.08 | 16.29 | 77.60 | 411.1 |
| 40 | 2013 | 11 | -13.2 | 16.26 | 77.20 | 409.9 |
| 39 | 2012 | 10.5 | -13.21 | 16.21 | 76.20 | 407.2 |
| 38 | 2011 | 10.1 | -13.2 | 16.15 | 73.50 | 406.5 |
| 37 | 2010 | 9.7 | -13.2 | 16.13 | 72.50 | 403.3 |
| 36 | 2009 | 9.3 | -13.19 | 16.14 | 71.70 | 409.9 |
| 35 | 2008 | 8.9 | -13.21 | 16.09 | 70.80 | 408.5 |
